# The Analysis of a Genome-Wide Association Study (GWAS) of Overweight and Obesity in Psoriasis

**DOI:** 10.3390/ijms23137396

**Published:** 2022-07-02

**Authors:** Anna Kisielnicka, Marta Sobalska-Kwapis, Dorota Purzycka-Bohdan, Bogusław Nedoszytko, Monika Zabłotna, Michał Seweryn, Dominik Strapagiel, Roman J. Nowicki, Adam Reich, Dominik Samotij, Justyna Szczęch, Dorota Krasowska, Joanna Bartosińska, Joanna Narbutt, Aleksandra Lesiak, Paulina Barasińska, Agnieszka Owczarczyk-Saczonek, Joanna Czerwińska, Jacek C. Szepietowski, Aleksandra Batycka-Baran, Rafał Czajkowski, Magdalena Górecka-Sokołowska, Lidia Rudnicka, Joanna Czuwara, Aneta Szczerkowska-Dobosz

**Affiliations:** 1 Department of Dermatology, Venereology and Allergology, Medical University of Gdansk, 80-210 Gdansk, Poland; dorota.purzycka-bohdan@gumed.edu.pl (D.P.-B.); bned@gumed.edu.pl (B.N.); monika.zablotna@gumed.edu.pl (M.Z.); rnowicki@gumed.edu.pl (R.J.N.); aneta.szczerkowska-dobosz@gumed.edu.pl (A.S.-D.); 2 Biobank Laboratory, Department of Molecular Biophysics, Faculty of Biology and Environmental Protection, University of Lodz, 90-237 Lodz, Poland; michal.seweryn@biol.uni.lodz.pl (M.S.); dominik.strapagiel@biol.uni.lodz.pl (D.S.); 3 Invicta Fertility and Reproductive Centre, Molecular Laboratory, 80-850 Gdansk, Poland; 4 Department of Dermatology, Institute of Medical Sciences, Medical College of Rzeszow University, 35-959 Rzeszow, Poland; adamandrzejreich@gmail.com (A.R.); dominik.samotij@gmail.com (D.S.); justyna.m.szczech@gmail.com (J.S.); 5 Department of Dermatology, Venerology and Paediatric Dermatology, Medical University of Lublin, 20-081 Lublin, Poland; dor.krasowska@gmail.com (D.K.); jbartosinski@gmail.com (J.B.); 6 Department of Dermatology, Pediatric Dermatology and Oncology Clinic, Medical University of Lodz, 90-419 Lodz, Poland; joanna.narbutt@onet.pl (J.N.); lesiak_ola@interia.pl (A.L.); paulina.barasinska@gmail.com (P.B.); 7 Chair and Department of Dermatology, Sexually Transmitted Diseases and Clinical Immunology, Collegium Medicum, University of Warmia and Mazury, 10-229 Olsztyn, Poland; agnieszka.owczarczyk@uwm.edu.pl (A.O.-S.); joannaj061@gmail.com (J.C.); 8 Department of Dermatology, Venereology and Allergology, Wroclaw Medical University, 50-367 Wroclaw, Poland; jacek.szepietowski@umed.wroc.pl (J.C.S.); aleksandra.batyckabaran@umed.wroc.pl (A.B.-B.); 9 Department of Dermatology and Venerology, Ludwik Rydygier Collegium Medicum in Bydgoszcz, Nicolaus Copernicus University in Torun, 87-100 Torun, Poland; r.czajkowski@cm.umk.pl (R.C.); magdalenagoreckaa@gmail.com (M.G.-S.); 10 Department of Dermatology, Medical University of Warsaw, 02-008 Warsaw, Poland; lidiarudnicka@gmail.com (L.R.); jczuwara@yahoo.com (J.C.)

**Keywords:** psoriasis, obesity, BMI, GWAS, gene polymorphisms

## Abstract

There is evidence that the concomitance of psoriasis and obesity may originate from the interplay between multiple genetic pathways and involve gene–gene interactions. The aim of this study was to compare the genetic background related to obesity among psoriatic patients versus healthy controls by means of a Genome-Wide Association Study (GWAS). A total of 972 psoriatic patients and a total of 5878 healthy donors were enrolled in this study. DNA samples were genotyped for over 500,000 single nucleotide polymorphisms (SNPs) using Infinium CoreExome BeadChips (Illumina, San Diego, CA, USA). Statistical analysis identified eleven signals (*p* < 1 × 10^−5^) associated with BMI across the study groups and revealed a varying effect size in each sub-cohort. Seven of the alternative alleles (rs1558902 in the *FTO* gene, rs696574 in the *CALCRL* gene, as well as rs10968110, rs4551082, rs4609724, rs9320269, and rs2338833,) are associated with increased BMI among all psoriatic patients and four (rs1556519 in the *ITLN2* gene, rs12972098 in the *AC003006.7* gene, rs12676670 in the *PAG1* gene, and rs1321529) are associated with lower BMI. The results of our study may lead to further insights into the understanding of the pathogenesis of obesity among psoriatic patients.

## 1. Introduction

Psoriasis is a chronic, inflammatory skin disease with a high worldwide prevalence reported to reach about 2.0% in the adult population [1]. Chronic plaque psoriasis constitutes the most common clinical manifestation of the disorder and is characterized by the formation of infiltrated plaques covered with silver scales. Nowadays, psoriasis is recognized as a complex disease with immunological, environmental, and genetic factors playing a pivotal role in its origin. It can occur at any age and has been reported both at birth and also in elderly people. Several large studies have described a bimodal psoriasis age of onset [2,3,4,5]. The mean age of onset for the first psoriasis occurrence can range from 15 to 20 years of age with a second peak occurring at 55–60 years. Type 1 begins on or before age 40 years; type II begins after the age of 40 years. The type I disease accounts for more than 75% of cases. Ref. [2] Immune system activation mediated by Th1 and Th17 lymphocytes, tumor necrosis factor-alpha (TNF-α), IL-17, IL-12, and IL-23, leads to the premature differentiation and hyperproliferation of keratinocytes resulting in the formation of scaling plaques. Furthermore, there is evidence of environmental factors, such as common infections, emotional stress, smoking, diet, and physical activity, having an influence on the disease course [6]. Recently, there have been a significant number of studies on the genetic background of psoriasis. The HLA-Cw*06:02 allele of the human leukocyte antigen gene (*HLA*) is located in the *PSORS-1* locus within the major histocompatibility complex (MHC) region on chromosome 6p21.3 and has been proven to be responsible for 30–50% of psoriasis genetic susceptibility [7]. A new era of Genome-Wide Association Studies (GWAS) enabled more than 80 single nucleotide polymorphisms (SNPs) involved in the multifactorial nature of psoriasis genetic background among Caucasian and Asian populations to be revealed. Most of them are located in proximity to genes coding for pathways responsible for skin barrier function (*LCE3B*, *LCE3C*), IL-23/Th17 signaling *(Il23R*, *IL23A,* and *IL12B*), IFN, and NF-κB signaling along with innate immunity (*TNFAIP3*, *TNIP1*, *NFKBIA*, *REL*, *TYK2, UBE2L3*, *CARD14*, *CARD6*, and *IFIH1*), adaptive immunity (*ERAP1*, *ZAP70*), and Th-2 activation response (*Il-4*, *Il-13*) [7,8,9].

The overproduction of the pro-inflammatory factors cascades in psoriasis results in their systemic release and circulation. In consequence, they exert pleiotropic effects, resulting in a greater prevalence of the psoriasis comorbidities in comparison to the general population, such as: obesity, hypertension, insulin resistance and diabetes, dyslipidemia, metabolic syndrome, or cardiovascular disease [10]. Among them, obesity seems to be a crucial driving force in their development, since the overabundance of adipose tissue is the source of manifold pro-inflammatory mediators—immunological factors (dendritic cells, cytokines, T-cells, macrophages, and chemokines), adipokines (adiponectin and leptin), chemokines and cytokines—which promote oxidative stress, endothelial dysfunction, and atherosclerosis [11,12]. There is evidence that psoriasis and obesity may originate from the elaborate interplay between multiple genetic pathways and gene-to-gene interactions. The results of a large cross-sectional study on Danish twins suggested a common genetic etiology for psoriasis and obesity [13]. Although rare monogenic variants associated with obesity result from highly infrequent single-gene mutations (such as *LEP*, *POMC*), some of them were also associated with polygenic obesity (e.g., *MC4R*, *FTO*). In the case of “common” multigenetic obesity, GWAS identified over 870 SNPs that determine only 3–5% of the disorder’s genetic background [14]. What is more, environmental and socio-economic aspects (for example, physical activity, nutrition, eating habits, smoking, and pollution) contribute in great measure to an individual’s disease susceptibility [14].

Lately, the genetic background of psoriasis has been investigated in detail. However, only little is known about the genetic susceptibility of psoriatic patients to overweight and obesity. Obesity among psoriatic patients may lead to significant clinical consequences, mainly early onset of the skin lesions, more severe psoriasis clinical course, reduced response to the systemic therapy, diminished life quality, and incidence of comorbidities leading to reduced life expectancy (chiefly due to cardiovascular events) [15,16,17]. The aim of this study was to investigate the genetic background of overweight and obesity among psoriatic patients.

## 2. Results

### 2.1. The Epidemiology of Overweight and Obesity among Psoriatic Patients and Control Group

**Figure 1:**
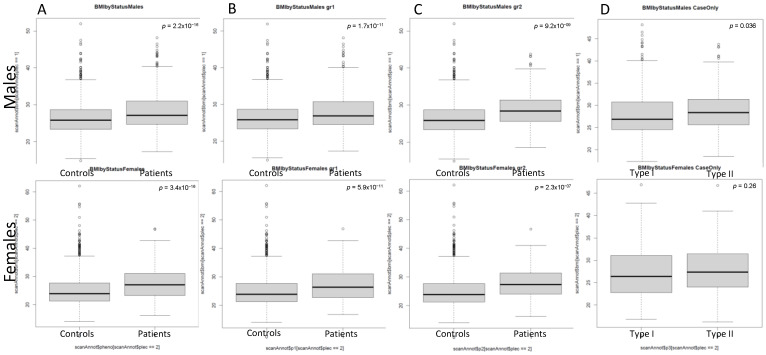
Boxplot of BMI (body mass index, kg/m^2^) among the study group (by gender): Panel (**A**–**C**) depicts the summary statistics of BMI in control group versus all psoriatic patients (**A**), type I psoriatic patients (**B**), and type II psoriatic patients (**C**), respectively. Panel (**D**) compares BMI of type I psoriatic patients vs. type II psoriatic patients. The central line in each box indicates the sample median, the boxes represent the 25th and 75th percentile; whiskers represent the confidence interval for the sample quantiles. Circles represent outliers. The *p* values for the Wilcoxon test are presented for every pair of boxplots.

Figure 1 presents the evidence for a greater predisposition of psoriatic patients to overweight and obesity than control donors. In general, psoriatic patients had statistically significant higher BMI values than the control group (*p* = 2.2 × 10^−16^ for males and *p* = 3.4 × 10^−16^ for females). There were statistically significant differences between type I and type II male psoriasis patients (*p* = 0.036); however, the discrepancies in BMI values were insignificant for female psoriatic patients (*p* = 0.26).

### 2.2. The Influence of Genetic Variants on the BMI across Different Study Groups

**Figure 2:**
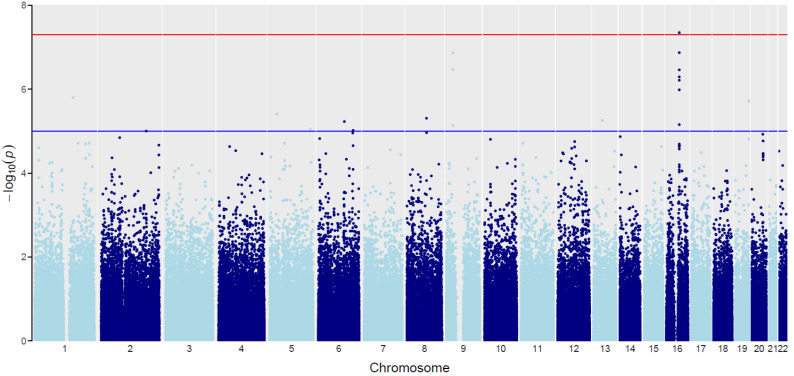
The “Manhattan” plot for GWAS interaction analysis of the BMI and psoriasis types association. Chromosomes are represented on the *x*-axis (black and grey dots). The −log_10_ (Joint.pval) is represented on the *y*-axis. Blue line indicates a suggestive association threshold (Joint.pval = 1 × 10^−5^). Red line indicates the genome-wide significant threshold (Joint.pval = 5 × 10^−8^).

Using a linear mixed model approach, we identified 135 SNPs with *p* < 1 × 10^−5^ (data presented in Supplementary Table S1). There were 56 SNPs mapped to the non-coding regions. Of the remaining 76 SNPs within the genes coding sequences, as many as 16 were located in the *FTO* gene, 3 SNPs were located in the *PTPRR* gene, and 2 SNPs each in *C1orf227 (SPATA45)*, *C5orf64*, *PAG1*, *RP11-2O17.2*, *RREB1,* and *TFCP2.* Using the FUMA GWAS method, we selected 11 loci in the coding and non-coding regions of the genome related to the body mass index in the study group (considering the division into type I and type II psoriasis) (Table 1; Figure 2). The distribution of genotype frequencies of 11 selected SNPs is presented in Supplementary Table S2. The regional association plots for the most interesting, statistically significant SNPs from GWAS results for the interaction effect between BMI and psoriasis are depicted in Supplementary Figure S1. For better presentation of our results, the box plots for interaction between SNP genotypes, BMI, and the tested group were also prepared (Supplementary Figure S2).

#### 2.2.1. Coding SNPs

As far as the coding SNPs are concerned, the lowest value of statistical significance for the entire study group in the context of BMI values was observed for rs1558902 (*p* = 4.47 × 10^−8^, *FTO* gene). Allele A carriers in the whole study group had an increased BMI of approximately 0.32 kg/m^2^. In the type I psoriasis group, the effect of the SNP on BMI was greater as it increased the BMI by about 0.87 kg/m^2^ in comparison to the group of healthy individuals.

In the entire study group (both control group and psoriatic patients), the effect of the rs1556519 (*ITLN2* gene) on the BMI index reduced the BMI values by 0.18 kg/m^2^. However, in the groups of patients with type I or II of psoriasis, it increased the BMI by 1.47 kg/m^2^ and 1.22 kg/m^2^, respectively, in comparison to the control group.

A comparable effect was observed for rs12972098 in the *AC003006.7* gene. People with type I and type II of psoriasis with the G allele had a higher BMI by 1.41 kg/m^2^ and 2.21 kg/m^2^, respectively, in comparison to the control group. Furthermore, the presence of the rs12972098 G allele among the whole study group decreased BMI by about 0.4 kg/m^2^.

The rs12676670 A allele in the *PAG1* gene lowered the BMI values in the entire study group (by about 0.31 kg/m^2^). However, it caused a significant BMI increase in the group of patients with type I psoriasis (by about 1.26 kg/m^2^) compared to the group of healthy people and only a slight increase in BMI in the group of patients with type II psoriasis (by 0.12 kg/m^2^).

Carriers of the rs696574 T allele of the *CALCRL* gene (*AC007319.1*) with psoriasis types I and II had a higher BMI by 1.3 kg/m^2^ and 2.28 kg/m^2^, respectively, compared to the control group. There was no effect on the BMI value among the carriers of the T allele throughout the study group (Gene-BMI Int = 0.016 kg/m^2^).

#### 2.2.2. Non-Coding SNPs

In the entire study group, carriers of the rs10968110 C allele had an increased BMI by about 0.23 kg/m^2^ compared to homozygous major carriers. The effect of the minor allele was visible in the group of patients with type I psoriasis, causing an increase in BMI by slightly more than 1.0 units of BMI compared to the control group (without psoriasis). For patients with type II psoriasis, the effect of the influence on BMI was the opposite and caused a decrease in BMI by 0.64 kg/m^2^ in this group (protective effect).

In general, allele T carriers of rs4609724 had an increased BMI of approximately 0.31 kg/m^2^ compared to those without this allele. The same association was also observed in the group of patients with type I psoriasis (increased BMI by about 0.68 kg/m^2^). However, the presence of the T allele among type II psoriasis patients reduced BMI by as much as 3.54 units (protective effect) as compared to the control group.

The genetic effect of the tested SNP rs4551082 was insignificant with an interesting interaction effect indicating differences in effect size between type I and II psoriasis (Gene-BMI Int pval = 0.00018). However, G allele carriers in the psoriasis type patient groups had a higher BMI (type I: 1.01 kg/m^2^ and type II: 0.97 kg/m^2^) compared to the control group, among which this allele increased BMI by only about 0.13 kg/m^2^ units.

In the whole study group, carriers of the G allele rs9320269 had an increased BMI by about 0.32 kg/m^2^. This allele had a protective effect among the whole patients’ groups (both type I and type II) in comparison to the controls—BMI was decreased by 1.09 kg/m^2^ for type I psoriasis and by 2.49 kg/m^2^ for type II.

On the other hand, carriers of the A allele rs2338833 among the type I psoriasis patients had a statistically higher BMI (by 1.55 kg/m^2^) compared to the psoriasis type II group (BMI increased only by 0.38 kg/m^2^). In the latter, the presence of the A allele did not exert any effect on the BMI values.

Finally, carriers of the C allele rs1321529 had a lower BMI value by 0.34 kg/m^2^ than those with the T allele. In the group of patients with psoriasis, carriers of the C allele among type I psoriasis patients had an increased BMI by 0.95 kg/m^2^ compared to the controls. Type II psoriasis patients had a lower BMI value by 1.36 kg/m^2^ (on average) as compared to the control group.

## 3. Discussion

In our study, we tested the genetic variants from Illumina Infinium CoreExome-24 BeadChips and evidence of differences between type I and type II psoriasis modified by body mass index. This application included both exonic and intronic, nonsense, and missense markers. Although the mitochondrial DNA (mtDNA) markers and sex chromosome variants were also tested on the microarrays, we excluded them from the statistical analysis to focus only on autosomal variants. The results of our study imply that there are significant interactions between 11 genetic polymorphisms and abnormal body mass index among psoriatic patients in general. Seven of them (rs1558902 in *FTO* gene, rs696574 in *CALCRL* gene, rs10968110, rs4551082, rs4609724, rs9320269, and rs2338833,) may cause an increase in BMI among all psoriatic patients and four genetic variants (rs1556519 in *ITLN2* gene, rs12972098 in *AC003006.7* gene, rs12676670 in *PAG1* gene, and rs1321529) may decrease the BMI value. However, considering the psoriasis types, only one SNP had an obesity-protective effect among type I psoriasis (rs9320269), while obesity-protective SNPs were more prevalent in the group of type II psoriasis (rs10968110, rs4609724, rs9320269, and rs1321529). Hitherto, our findings were not described before by other researchers, which implies that further insight may lead to the understanding of the pathogenesis of overweight and obesity among psoriatic patients.

In our study, the A allele of rs1558902 in the *FTO* gene was associated with considerably greater BMI values, especially among type I psoriasis patients. The *FTO* gene encodes 2-oxoglutarate-dependent nucleic acid demethylase, which is a protein responsible for the DNA restoration and processes regulating energy homeostasis [18]. Although its precise physiological function is yet undiscovered, the *FTO* mRNA is highly expressed in the hypothalamic nuclei that constitute the organism’s homeostasis regulating region [18]. Furthermore, genome-wide association studies have revealed that the *FTO* gene region is linked to obesity-related traits such as BMI value, hip circumference, and body weight [19]. Manifold studies showed that the strongest association of the *FTO* gene with overweight and obesity involved mainly A and G risk alleles of the following two SNPs: rs9930609 and rs9930506 [20,21,22,23]. Coto-Segura et al. found that among the psoriatic patients’ cohort of Spanish origin (European descent), the rs9930506 of the *FTO* gene was linked to higher BMI values, and thus greater obesity risk. However, there was no association with psoriasis disease activity. The authors concluded that psoriatic patients homozygous for the SNP’s risk allele had the highest risk of obesity [24]. Moreover, Tupikowska-Marzec et al. conducted a study among Polish psoriatic patients of the Lower Silesia region regarding the *FTO* gene rs9939609 variant (A and T alleles). The A allele was identified to be the “risk allele” related to not only elevated BMI and hip and waist circumference measurements, but also to a higher PASI index (Psoriasis Area Severity Index), CRP (C-reactive protein) values, and serum insulin concentrations. The authors, however, did not find the allele’s greater prevalence among the psoriatic patients’ group [25]. Finally, in the context of the A allele of the *FTO* gene (rs1558902) investigated in our study, Ślęzak et al. evaluated its influence on polish male patients with metabolic syndrome. Although a strong association of the AA genotype with BMI, WHR, and dyslipidemia (mainly cholesterol and triglyceride levels) was revealed in comparison to healthy controls, the researchers did not confirm the significance of the *FTO* gene polymorphisms among the risk factors of the metabolic syndrome [26]. All in all, further insight is necessary to elaborate on the *FTO* gene’s role in the mechanism of obesity pathogenesis and metabolic disturbances among psoriatic patients. Possible links may include the interplay between the intronic SNPs of the *FTO* gene and proximal genes, alterations of the epigenetic processes, or the interactions with dopaminergic neurotransmission and ghrelin-mediated signaling [27,28].

The *CALCRL* gene (calcitonin receptor-like receptor) codes for the G-protein coupled receptor linked to adrenomedullin—a peptide involved in the regulation of manifold physiological pathways, particularly linked to cardiovascular and lymphatic systems [29,30]. Harmancey et al.’s study revealed that adrenomedullin may play a role in adipogenesis as an antiadipogenic agent due to inhibited adipocyte differentiation in an adrenomedullin-depleted synthesis environment [31]. On the other hand, Li et al. investigated that adrenomedullin may be classified as an adipokine family protein, owing to its elevated levels both in plasma and adipose tissue among of obese population [32]. The GWAS analysis performed by Aguilera et al. showed that the *CARCL* gene expression was downregulated in visceral adipose tissue of obese prepubertal children [33]. Similar results were described by Baranova et al. among the adult population and, therefore, may suggest the explanation of the adrenomedullin overabundance in obesity [34]. To the authors’ knowledge, currently, there are no studies evaluating the role of the *CARCRL* gene among psoriatic patients.

The omentin-2 (encoded by *ITLN2* gene—also known as intelectin), along with its homologous isoform omentin-1, is chiefly expressed in the omental adipose tissue. Since the omentin-1 homolog plays a pivotal role in humans, the exact omentin-2 role remains ambiguous [35]. Human omentin is a newly identified adipokine with an anti-inflammatory potential, which exhibits an inverse correlation with BMI and WHR [36]. Furthermore, the preliminary studies of omentin significance among psoriatic patients demonstrate its decreased levels, which promotes the PASI increase and overabundance of the fatty tissue [37,38,39]. Therefore, in the context of our study, it could be a possible explanation for the *ITLN2* gene BMI-increasing effect among the psoriatic patients’ group.

The *PAG1* gene codes for the type III transmembrane adaptor protein involved in the regulation of T cell activation [40]. Wu et al. used target capture sequencing to describe a correlation of the genetic variants of the *PAG1* region with obesity among the Northern Han Chinese population [41]. In our study, the A allele of the *PAG1* gene was associated with a significant increase in BMI among psoriasis type I patients. However, more research is needed to state the gene polymorphism function in the pathogenesis of psoriasis.

Our study provided a number of other gene polymorphisms, which were found to vary in prevalence among overweight and obese psoriatic patients (such as: the *AC003006.7* gene, rs10968110, rs4551082, rs4609724, rs9320269, rs2338833, and rs1321529). However, their up-to-date biological function and clinical importance remain unknown, particularly in the psoriasis disease context.

All in all, the results provided in our research constitute an interesting preliminary foundation for further investigation. With the presented study, we had the opportunity to evaluate the interaction effect between SNPs, BMI, and two psoriasis subtypes (type I and type II), which have not been tested to date. To date, various possible mechanisms linking obesity to dermatitis due to the functional changes in adipose tissue have been tested [42]. Excess skin adipose tissue causes the secretion of pro-inflammatory cytokines and hormones. Cytokines such as tumor necrosis factor-alpha (TNFα) and interleukin 6 (IL-6) are directly involved in the pathology of psoriasis and are targets of some highly effective therapies. Leptin may increase the proliferation of keratinocytes and the secretion of pro-inflammatory proteins that are characteristic of psoriasis, while the secretion of adiponectin, supposedly anti-inflammatory, is reduced in obesity [12].

Some likely limitations of our research must be considered. Firstly, the data need further evaluation and confirmation in larger prospective studies. Functional analysis of mapped genes and their validation in an in vitro study on the skin cells and adipose tissue physiology are also needed. What is more, evaluation of the proinflammatory pathways’ interplay, oxidative stress, and lipid concentrations in obese psoriatic patients may lead to further insight into its common pathogenesis. It is also worth emphasizing the fact of reduced physical activity among psoriatic patients due to the general stigma of psoriasis disease, which discourages many patients from undertaking any exercises and, thus, might have a direct impact on the development of obesity [43]. In a study by Leino et al., 23.7% of psoriatic patients reduced their sporting activities and 30.2% of them ceased it completely due to skin disease [44]. Psoriasis-specific factors such as: disease severity, skin sensitivity, clothing choice, and treatment contribute to exercise avoidance [45]. We were also unable to include in our statistical analysis many other significant factors, such as level of physical activity, nutrient intake, socioeconomic status, or other environmental covariates that might influence results. As they were not included in questionnaires for the control group, we were not able to create a dataset for gene–environment (G × E) interaction analyses. To the best of our knowledge, G × E interaction analyses remain challenging, even for the meta-analysis research of more than 200,000 individuals, in which, so far, only 12 loci have been identified, the effects of which on obesity are attenuated or exacerbated by non-genetic factors [46]. In the future, to define the impact of our findings, firstly, a greater number of study participants should be included and high-resolution fine-mapping analysis should be also performed. Furthermore, little is known about the gene–environment interaction in psoriasis so far, so this is still an area to be explored.

## 4. Materials and Methods

### 4.1. Patient Group

The patient’s cohort was recruited between 2010 and 2019 from individuals of Polish origin (Caucasian population) with chronic plaque psoriasis. The diagnosis was confirmed by clinical examination or histology. The enrolment process was carried out through dermatology wards and outpatient clinics in Poland (Central Europe). The procedure involved: (1) detailed demographic and clinical data collection; (2) dermatological skin examination; (3) body weight and height measurement; and (4) taking peripheral blood samples for DNA isolation, biobanking, and genetical analysis by qualified medical personnel. Each participant provided written informed consent. The study received approval from the Bioethics Committee for Scientific Research.

A total of 1179 psoriatic patients were reviewed. Of them, 972 patients with a complete dataset collection were included in the study. The cohort comprised of 353 (36.24%) women (mean BMI = 27.36 ± 5.76 kg/m^2^, mean age 49.02 years ± 16.07, range 11–85 years) and 619 (63.75%) men (mean BMI = 28.09 ± 4.98 kg/m^2^, mean age 45.5 years ± 14.95, range 15–93 years). The severity of the skin lesions was measured using the Psoriasis Area Severity Index (PASI), mean value PASI = 12.60 ± 9.40). Detailed data on the patient group analysis are presented in Supplementary Table S3.

### 4.2. Control Group

A total of 5878 donors with their genetic data were enrolled in this study. The control donors (Caucasian population, Central Europe) were recruited between 2010 and 2012 as a part of the TESTOPLEK research project and registered as POPULOUS collection at the Biobank Lab of The Department of Molecular Biophysics of The University of Łódź, Poland [47]. The exclusion criteria involved: psoriasis, current or past history of malignancy (including myeloid disorders), or bone marrow transplantation. The study was approved by The University of Łódź’s Review Board.

This control group consisted of 3001 (51.1%) females (mean BMI = 24.94 ± 5.06 kg/m^2^, mean age 43.8 ± 13.3 years, range 20.0–77.0 years) and 2877 (48.9%) males (mean BMI = 26.22 ± 4.17 kg/m^2^, mean age 43.0 ± 15.05 years, range 20.0–77.0 years).

### 4.3. DNA Isolation

For the samples from Populous collection, procedures concerning DNA extraction and sample processing have previously been described [48,49].

For the group of psoriatic patients, genomic DNA was extracted from whole blood using the MagNa Pure LC 2.0 (Roche, Basel, Switzerland). NanoDrop 1000 (Thermo Fisher Scientific Inc., Waltham, MA, USA) and Qubit 2.0 (Thermo Fisher Scientific Inc., Waltham, MA, USA) were used to determine DNA quality and concentrations. Afterward, DNA samples underwent PCR reaction for sex verification [50].

### 4.4. Microarrays Analysis

All DNA samples were genotyped using Infinium CoreExome microarrays. Array genotyping was performed according to standard Illumina protocols. Microarray scan data were then converted to genotypes using Genome Studio 2.0. Standard microarray quality control protocols, such as filtering on sample call rates (>0.94) or SNP genotyping rates (10%GenCall > 0.4), were executed. The results were exported from GenomeStudio using PLINK Input Report Plug-in v2.1.4 by forward strand.

### 4.5. Statistical Methods

Genetic data were filtered, thus only variants with minor allele frequencies above 5% were included. Subsequently, the Hardy–Weinberg equilibrium was tested and SNPs with a *p*-value below 0.05 were excluded from the analysis of association.

The genetic relatedness matrix was estimated using the KING-robust method available in package SNPRelate in R. Furthermore, this matrix was used by the PCAiR method (Principal Components Analysis in Related Samples) implemented in the GENESIS package to evaluate genetic diversity among study participants and evaluate the genetic principal components used in further analysis as covariates. The core genetic analysis based on linear mixed models was performed as implemented in package GENESIS in R. In the absence of family assignment, all individuals were treated as a single group; thus, the random effect associated with the correlation matrix associated with individuals’ trait values is reflected by the genetic relatedness matrix (kinship). We used BMI as the dependent variable and independent variables reflected by the principal components estimated from PCAiR (the first five components were tested to evaluate robustness) and associated with the study group (one control group and two groups of affected individuals). The main effect of each variant was tested for significance using the score test. In addition, a 2df model was considered, where the interaction between each SNP and the study group was introduced. In such a case, the estimated effects of interactions correspond to contrasts between the baseline effect (effect of SNP in the control group) and the effect of SNP in the study group of interest. Variants with a joint 2df *p*-value below 10^(−5)^ were reported in the results.

## 5. Conclusions

The overabundance of fatty tissue among psoriatic patients has a multifactorial origin and the interplay between genetic factors, lifestyle, diet, and environmental influence should be considered. The results of our study imply that there is an association between 11 genetic polymorphisms and abnormal body mass among psoriatic patients in general. Thus, potential directions of further genetic research concerning the etiology of psoriasis as a systemic disease associated with an increased risk of obesity were indicated. These findings may lead to insights into the understanding of the pathogenesis of obesity among psoriatic patients. The possible practical implications and importance of our as well as future detailed genetic studies include identifying patients with an increased risk of obesity among the psoriasis population. Early patients’ screening and education leading to prevention and treatment would decrease cardiovascular risk and, hence, a reduction in the mortality rate in psoriasis. However, more detailed research into gene–environment interactions in the pathogenesis of psoriasis and obesity would also be beneficial and a promising area to be explored.

## Figures and Tables

**Table 1:** GWAS results for the interaction effect between body mass index (BMI), single nucleotide polymorphisms (SNPs), and type I and II of psoriasis *.

SNP ID	Chr	Position	Gene-BMI Int: All	Gene-BMI Int: Type I	Gene-BMI Int: Type II	Gene-BMI Int Pval	Joint.Pval	Overlapped Gene	Gene Function
rs1558902	16	53803574	0.318627	0.873456933	0.29827713	0.00269419	4.47 × 10^−8^	*FTO*	Protein coding
rs10968110	9	27792965	0.225899	1.07044773	−0.645818448	5.95 × 10^−5^	1.35 × 10^−7^	None	None
rs1556519	1	160917902	−0.18039	1.477266915	1.211225346	3.73 × 10^−7^	1.58 × 10^−6^	*ITLN2*	Protein coding
rs12972098	19	58249304	−0.40813	1.418581983	2.2106271	2.04 × 10^−6^	1.90 × 10^−6^	*AC003006.7*	Protein coding
rs4551082	5	29620270	0.134778	1.007787695	0.972212178	0.000176593	3.87 × 10^−6^	None	None
rs12676670	8	81981261	−0.03538	1.264637409	0.120877962	3.50 × 10^−6^	4.93 × 10^−6^	*PAG1*	Protein coding
rs4609724	13	55666639	0.308242	0.677809579	−3.537824099	1.52 × 10^−5^	5.56 × 10^−6^	None	None
rs9320269	6	109032557	0.320152	−1.09262589	−2.489035704	3.04 × 10^−6^	5.90 × 10^−6^	None	None
rs2338833	5	169512063	0.0088	1.555408166	0.378795107	1.18 × 10^−5^	8.91 × 10^−6^	None	None
rs1321529	6	145601560	−0.34021	0.9503797	−1.359245974	0.00015023	9.59 × 10^−6^	None	None
rs696574	2	188228516	0.01579	1.031042583	2.283444931	1.53 × 10^−5^	9.96 × 10^−6^	*CALCRL*	Protein coding

* SNP ID—single nucleotide polymorphism identificator; Chr—chromosome; Position—position in the GRCh37 reference genome; Gene-BMI Int—the effect size estimate for the genotype term in the tested group (all, type I, and type II refer to the entire case-control statistics, type I of psoriasis vs. controls, and type II of psoriasis vs. controls, respectively); Gene-BMI Int pval—the Wald *p*-value for the test of all genotype interaction effect; Joint.pval—the Wald *p*-value for the joint test of the genotype term and all of the genotype interaction effects.

## Data Availability

The data that support the findings of this study are available from the corresponding author upon reasonable request.

## Supplementary Materials

The following supporting information can be downloaded at: https://www.mdpi.com/article/10.3390/ijms23137396/s1.
