# Supplementary material for: The Analysis of a Genome-Wide Association Study (GWAS) of Overweight and Obesity in Psoriasis
Source: Int J Mol Sci. 2022 Jul 2;23(13):7396. doi: 10.3390/ijms23137396 (PMC9266424; doi:10.3390/ijms23137396)

In this supplementary file we present the regional association plots ("Zoom" plot) for the most interesting, 11 statistically significant SNPs from GWAS results for the interaction effect between body mass index (BMI) and psoriasis. The top SNP on every plot is the SNP of interest and has the smallest p value in the region. SNPs are colored based on their correlation (LD).

1. Coding SNPs:
   1.1. **rs1558902**, *FTO*

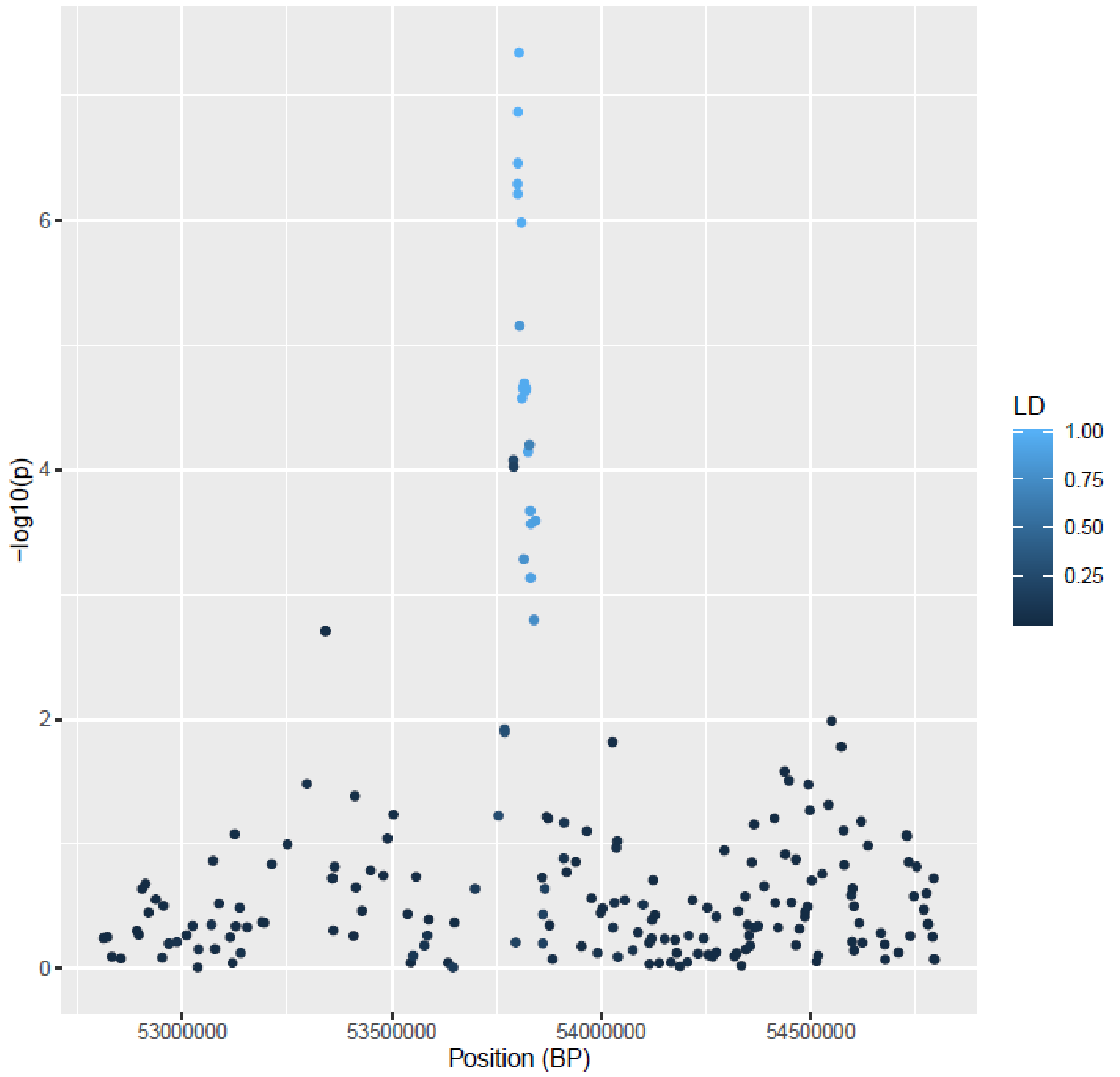

1.2. **rs1556519**, *ITLN2*

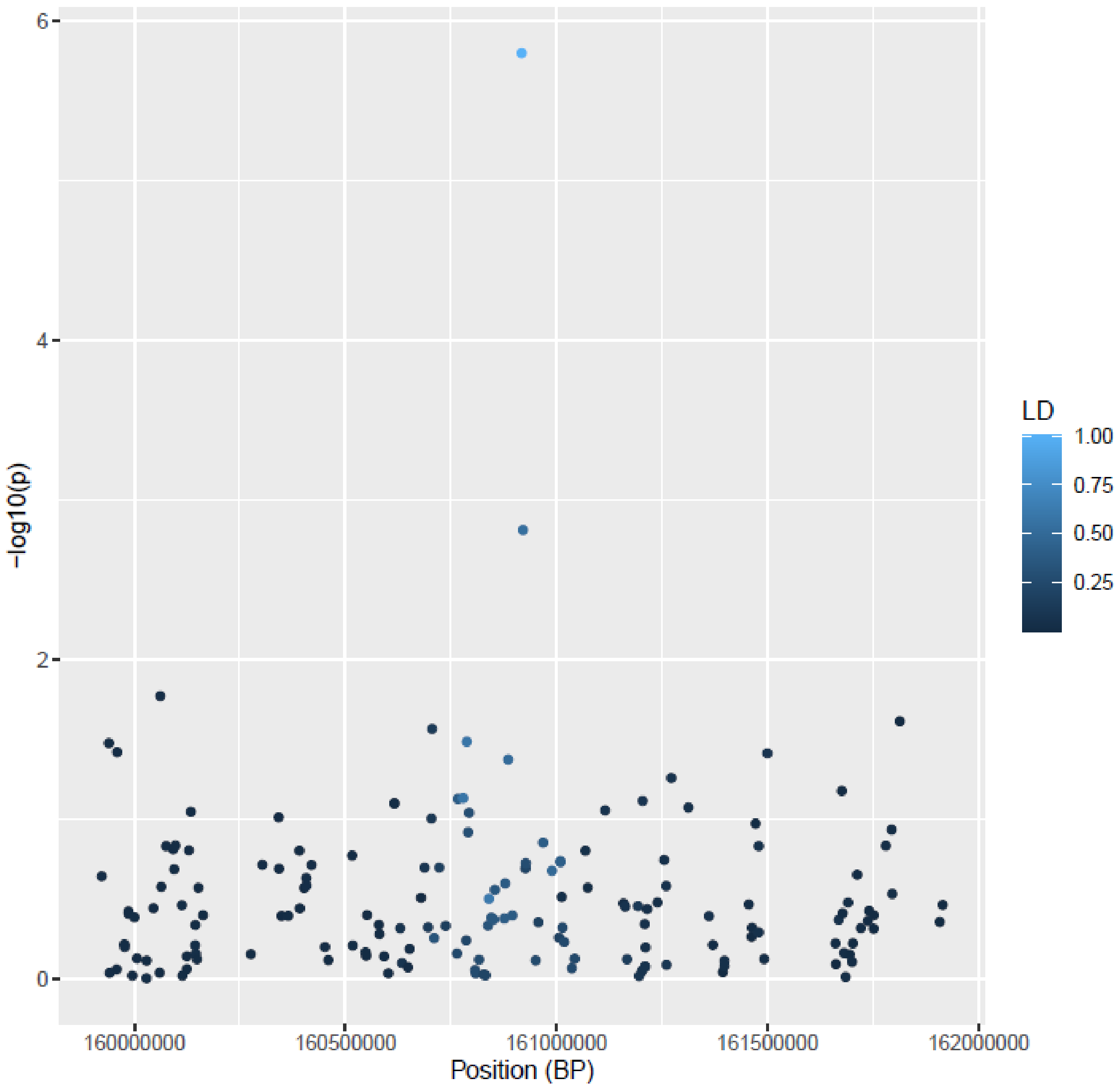

1.3. **rs12972098**, *AC003006.7*

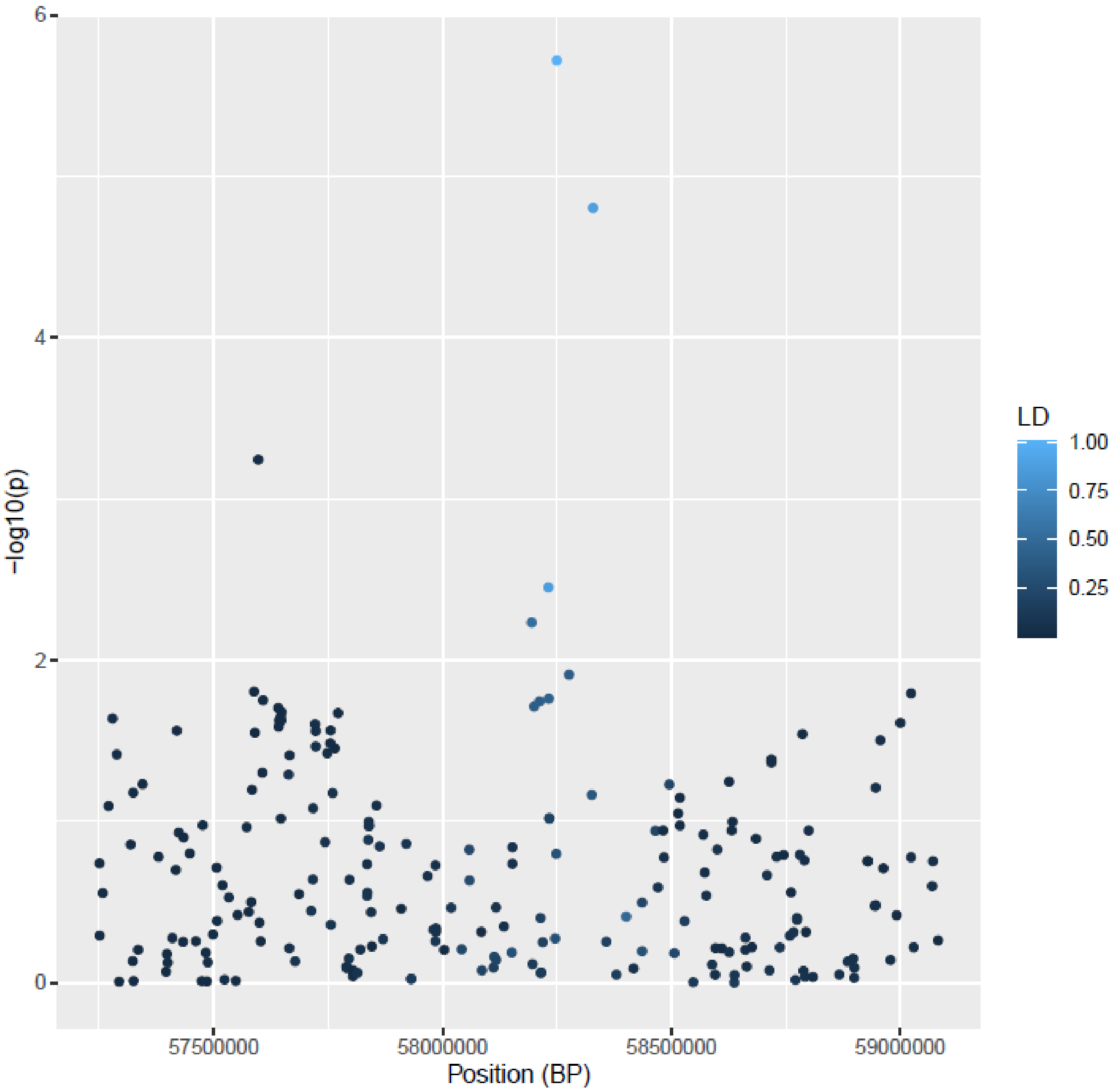

1.4. **rs12676670**, *PAG1*

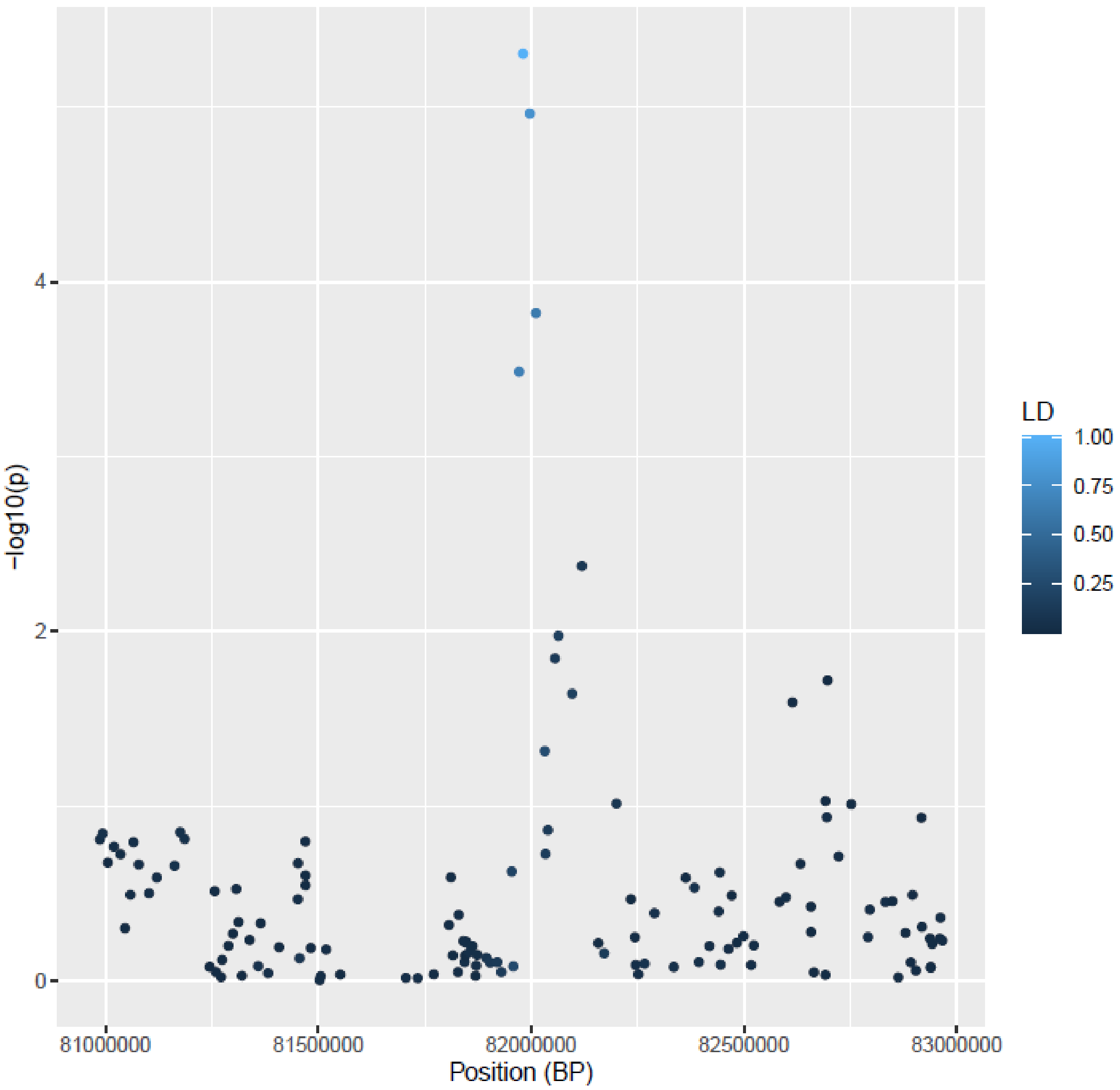

1.5. **rs696574**, *CALCRL*

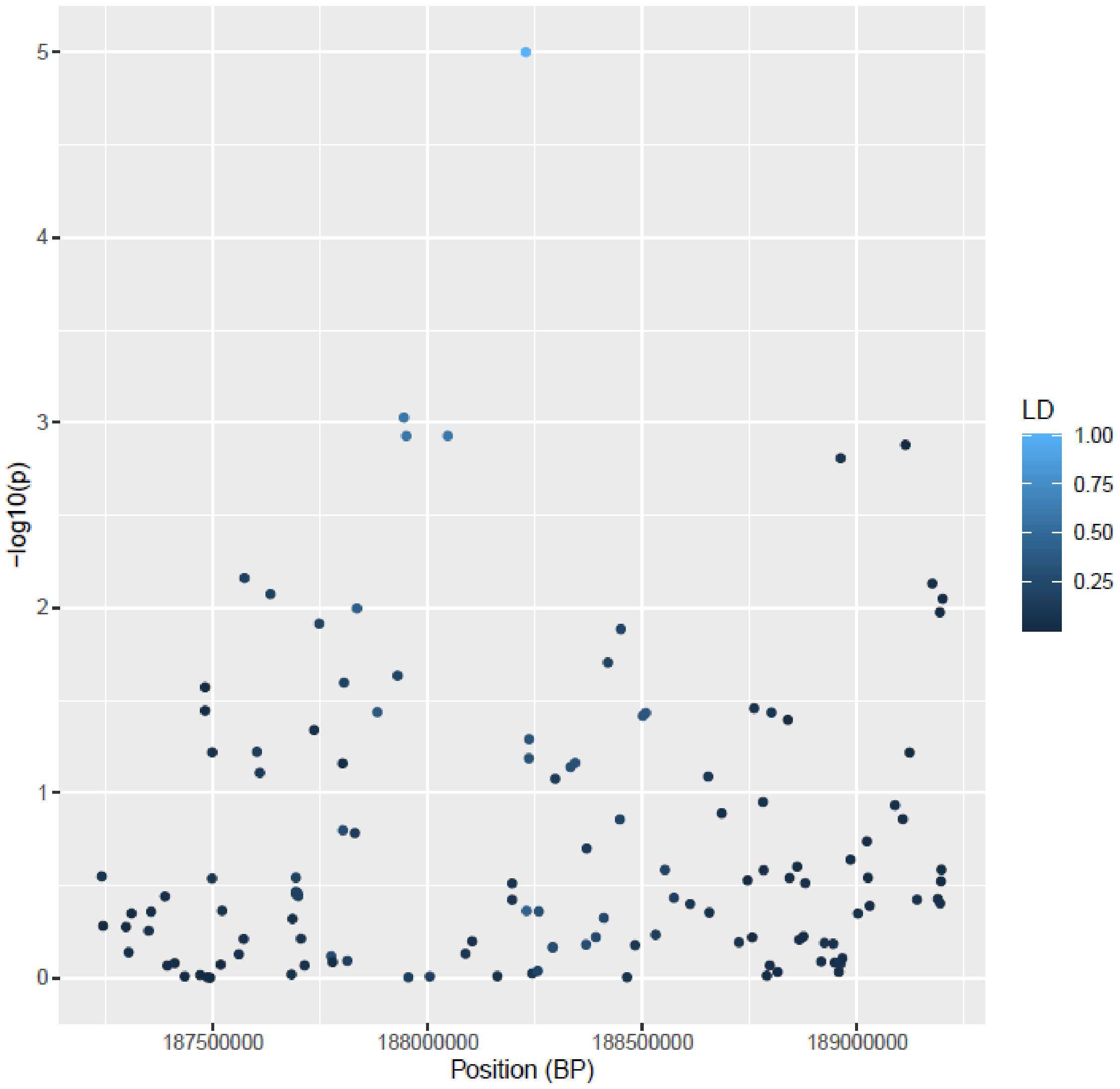

2. Non-coding SNPs:

2.1. **rs10968110**, chr 9, 27792965 bp

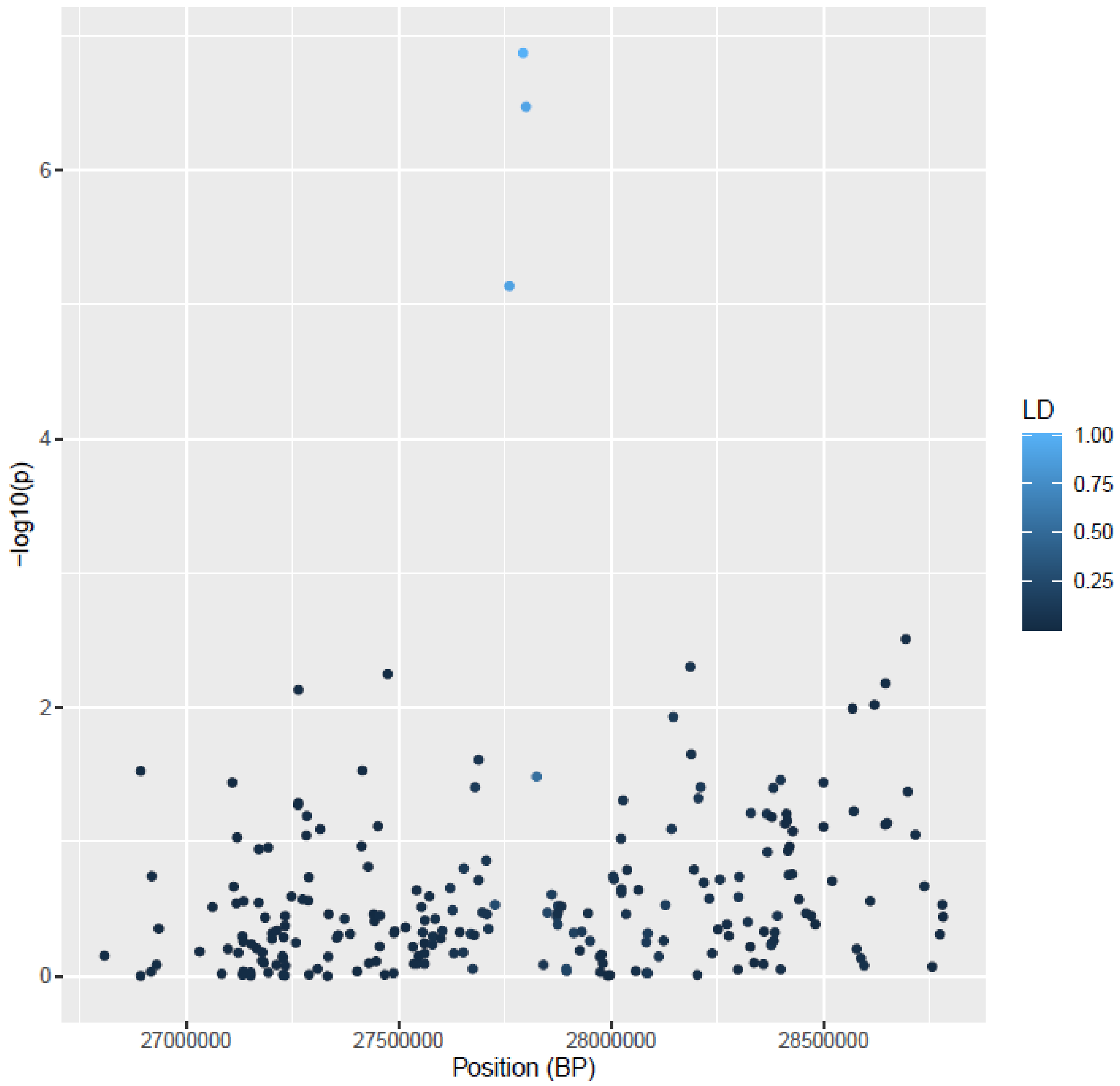

2.2. **rs4609724**, chr 13, 55666639 bp

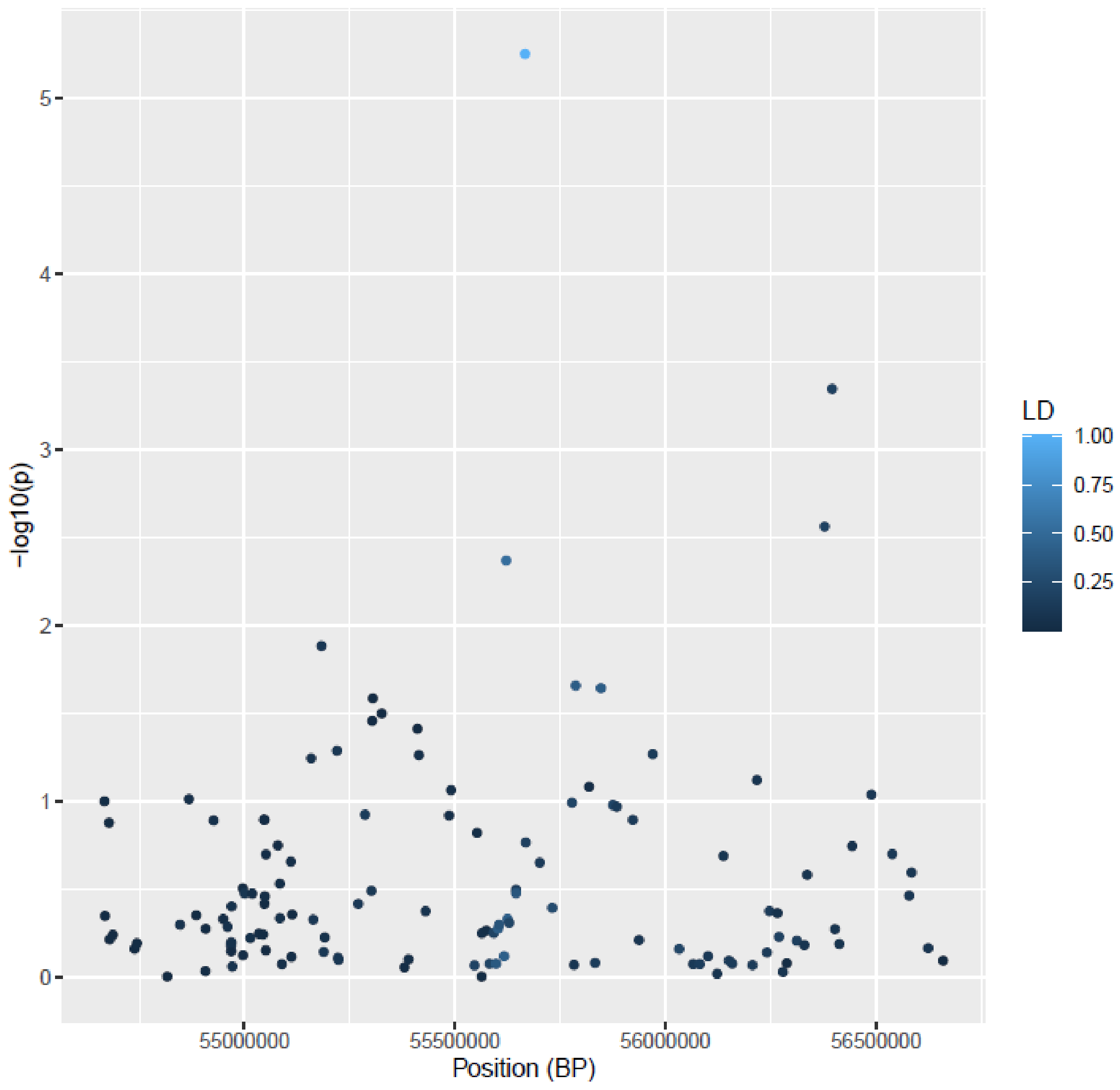

2.3. **rs4551082**, chr 5, 29620270 bp

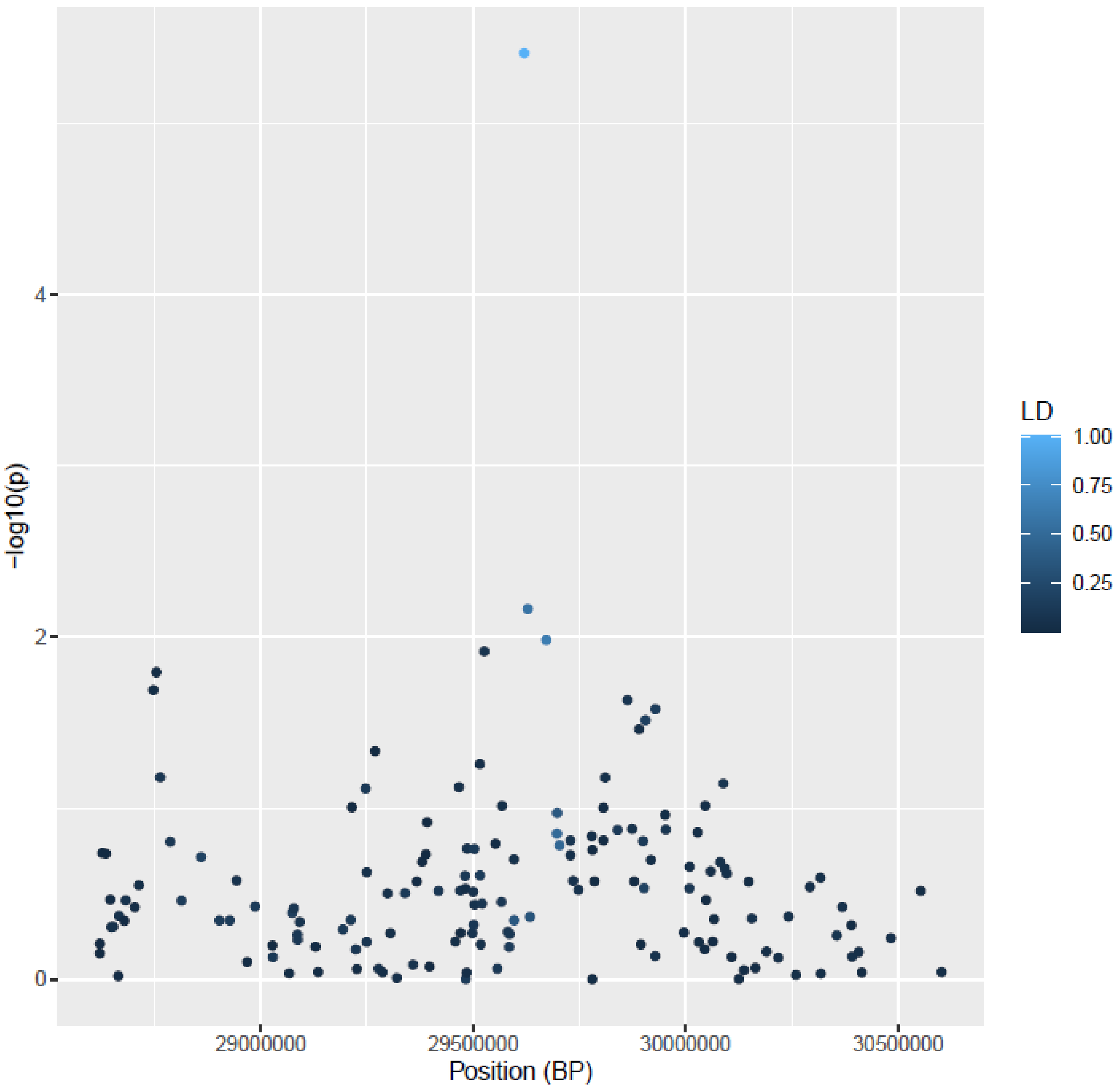

2.4. **rs9320269**, chr 6, 109032557 bp

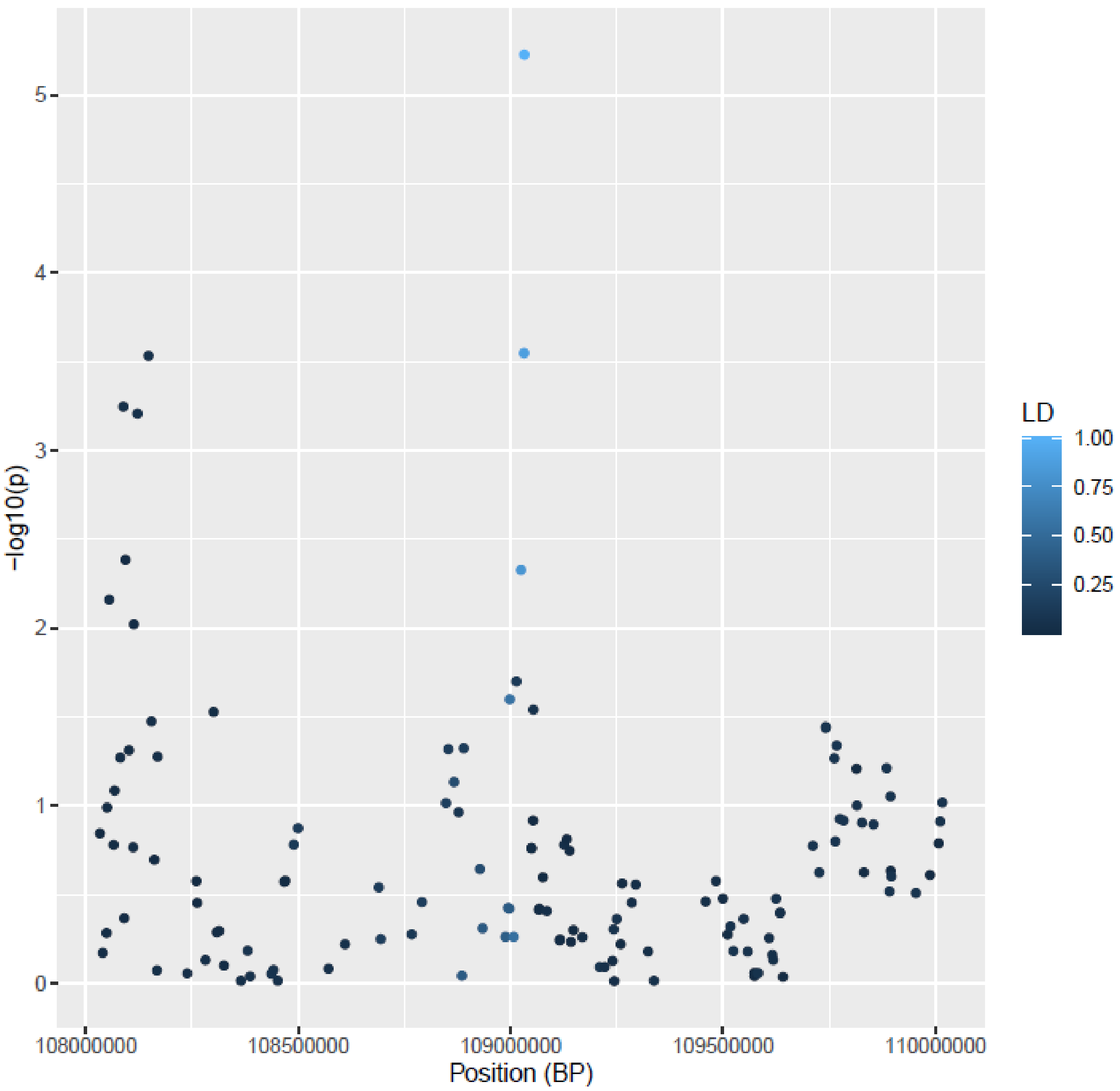

2.5. **rs2338833**, chr 5, 109032557 bp

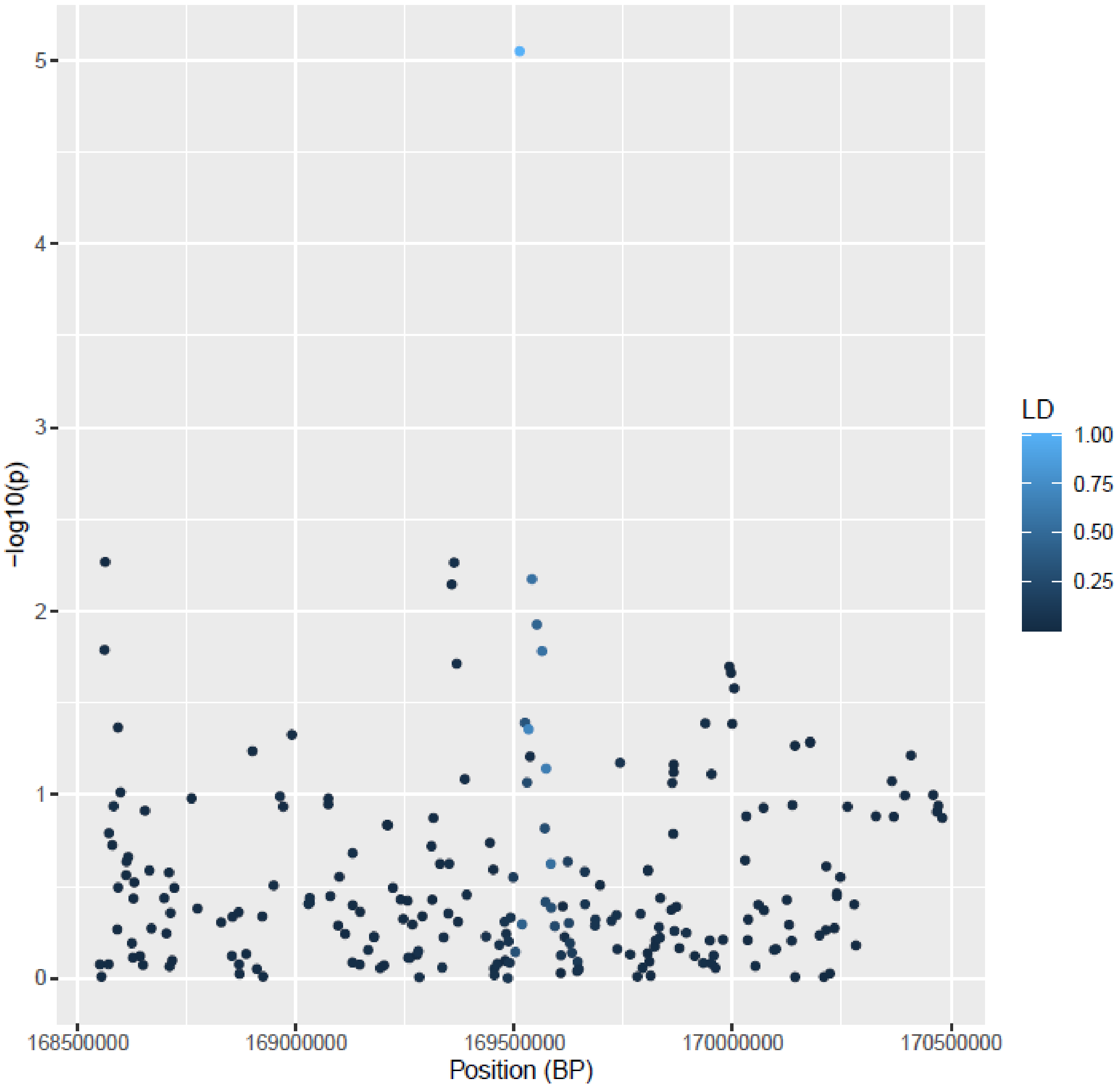

2.6. **rs1321529**, chr 6, 145601560 bp

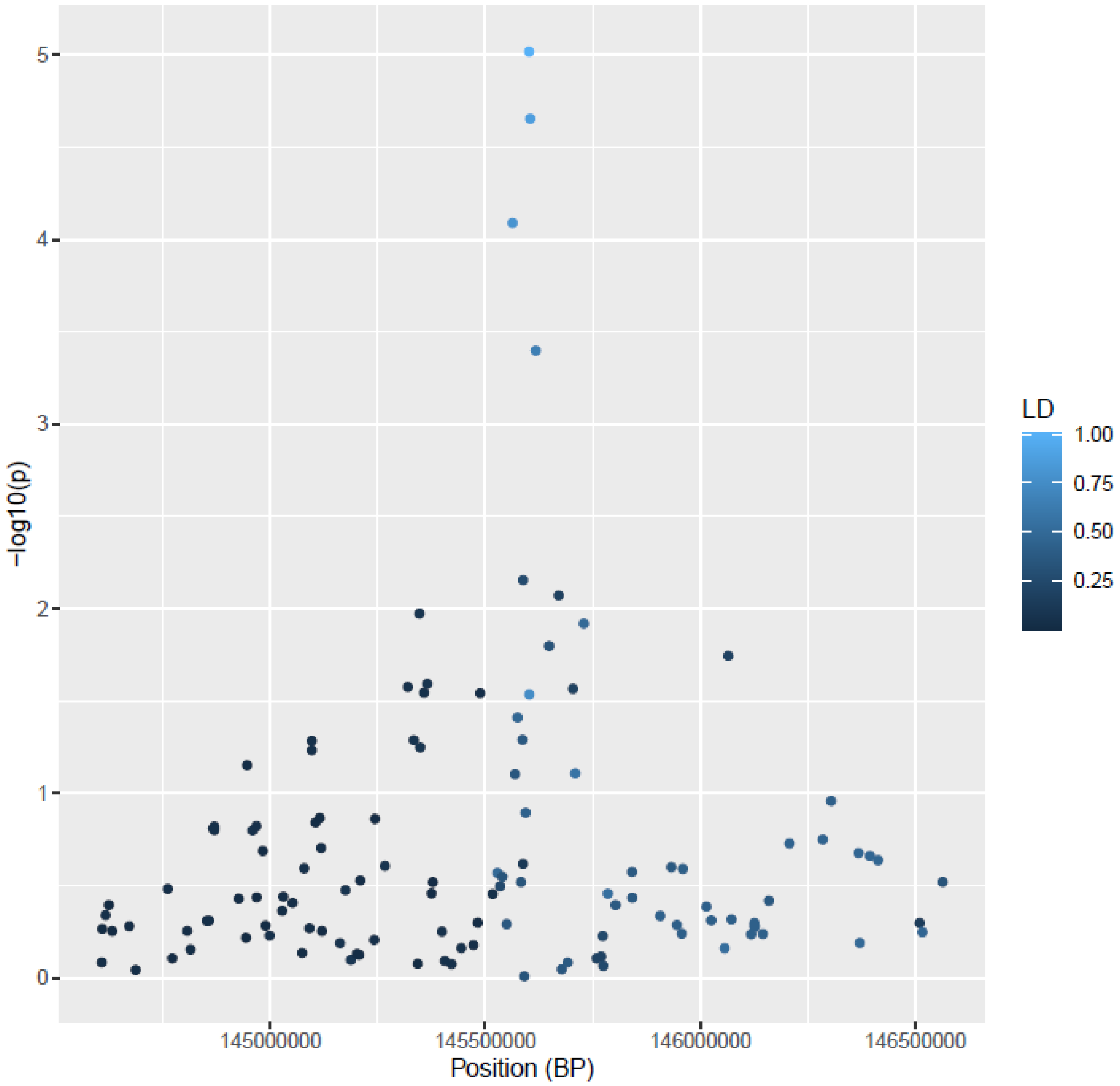

Supplement: Supplementary file 1 [file ijms-23-07396-s001.zip › Supplementary Figure S1.pdf]
