# Supplementary material for: The Analysis of a Genome-Wide Association Study (GWAS) of Overweight and Obesity in Psoriasis
Source: Int J Mol Sci. 2022 Jul 2;23(13):7396. doi: 10.3390/ijms23137396 (PMC9266424; doi:10.3390/ijms23137396)

With this supplementary file we present boxplots for BMI across study groups (0=control, 2=type I psoriasis, 3=type II psoriasis) and SNP genotypes (0=homozygote major, 1=heterozygote, 2=homozygote minor). The lines represent median, the hinges stand for quartiles and whiskers are defined by the limits of the confidence interval for the quartiles.

1. Coding SNPs:
   1.1. **rs1558902**, *FTO*

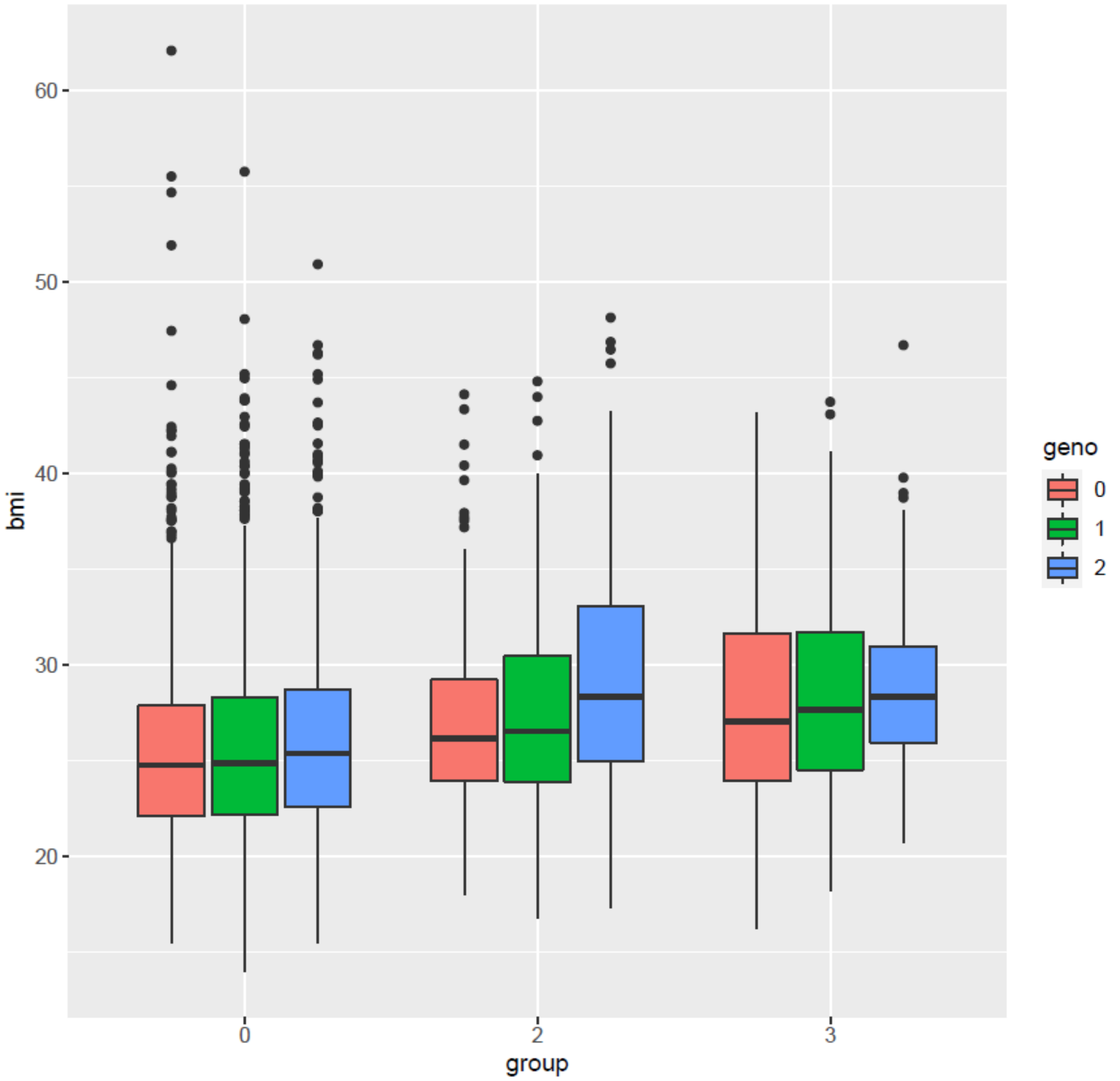

1.2. **rs1556519**, *ITLN2*

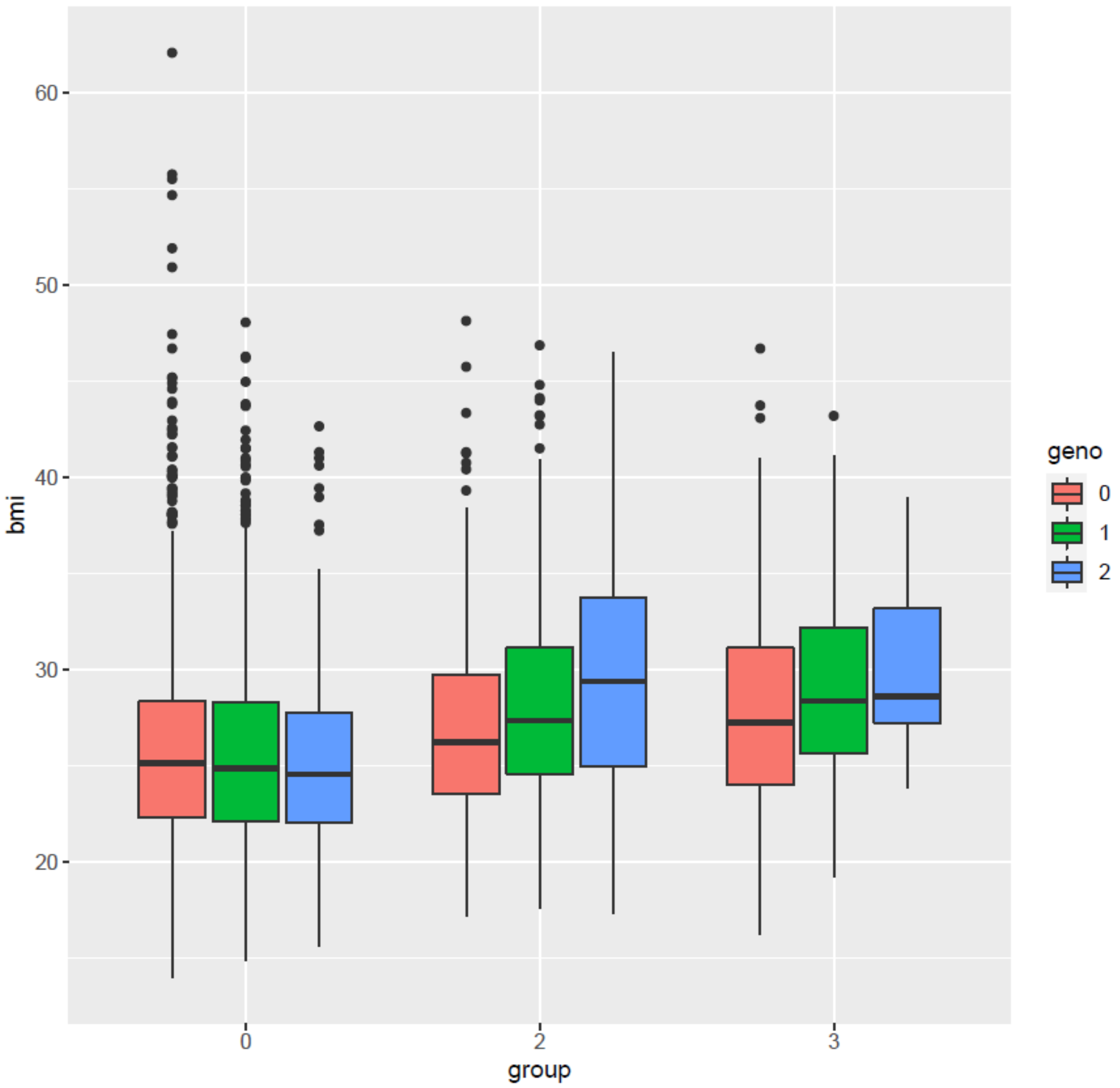

1.3. **rs12972098**, *AC003006.7*

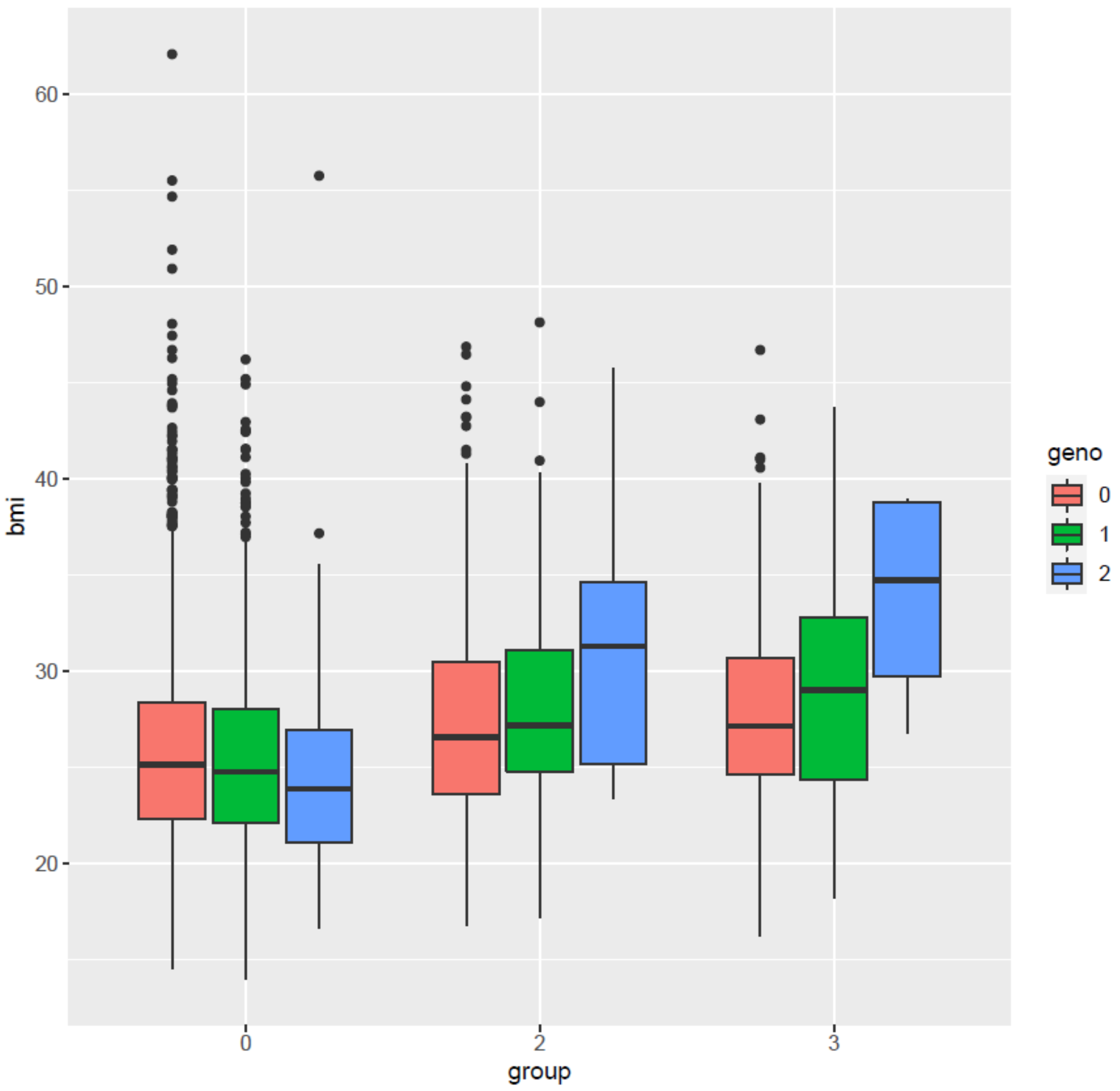

1.4. **rs12676670**, *PAG1*

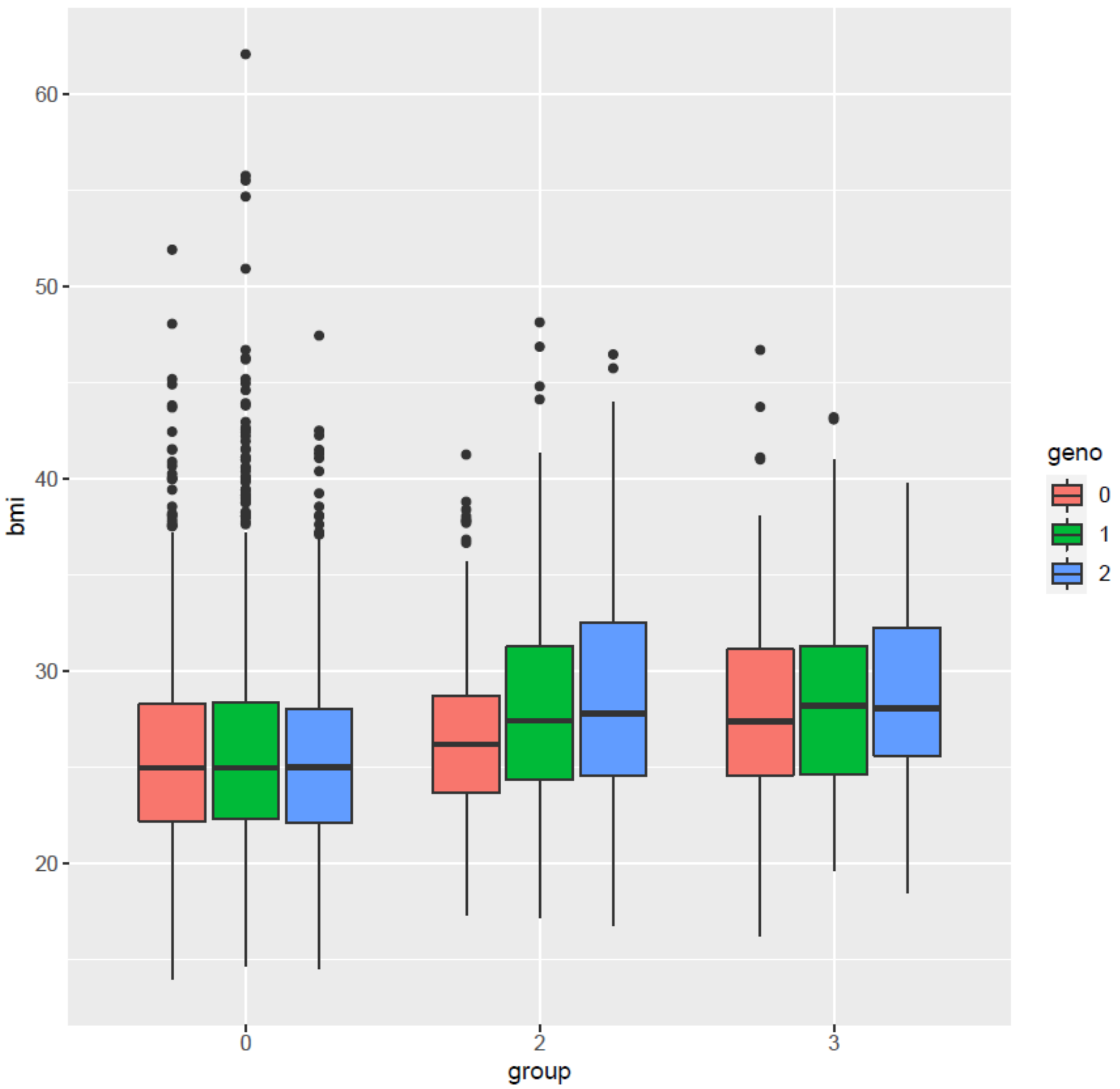

1.5. **rs696574**, *CALCRL*

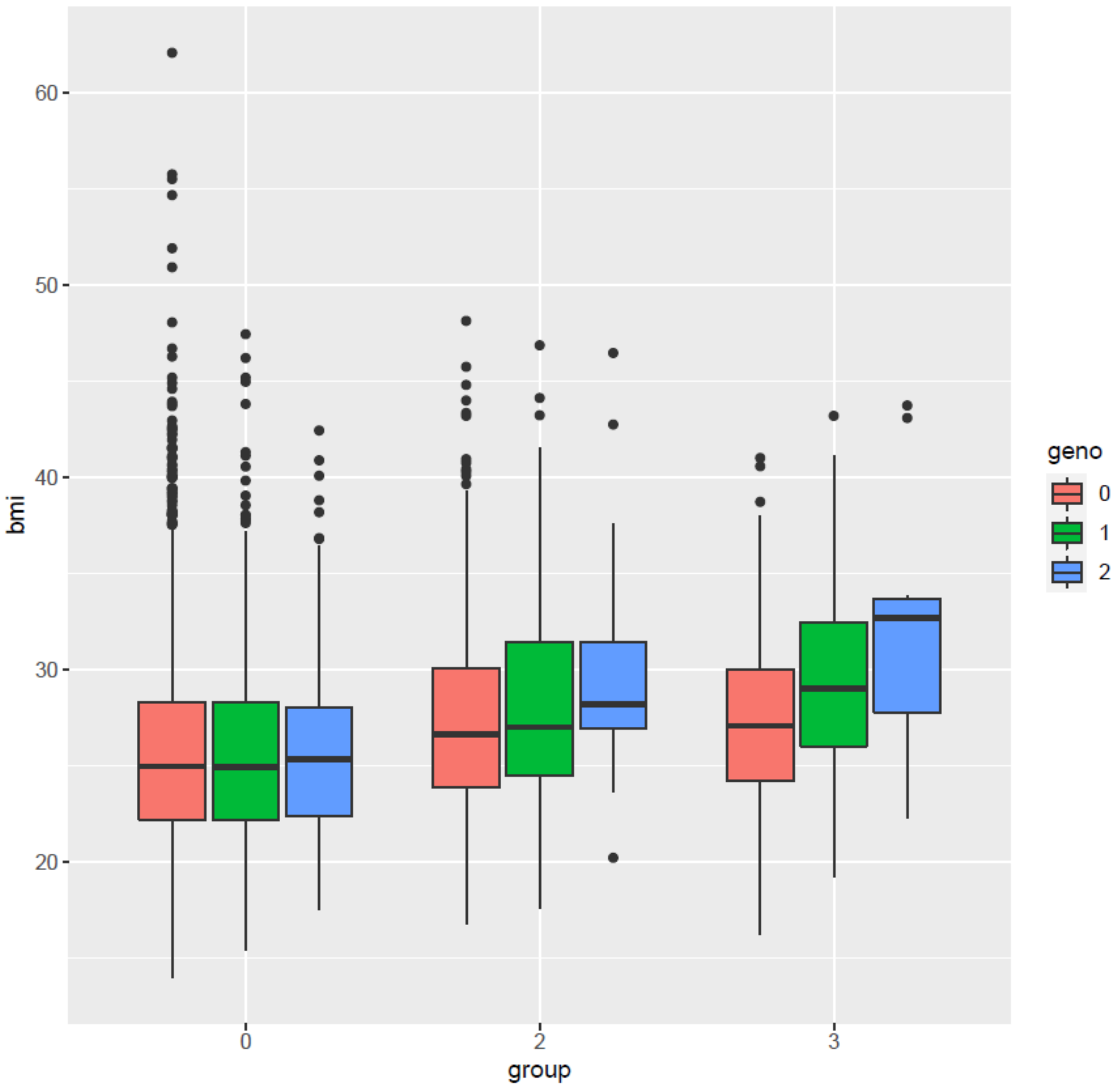

2. Non-coding SNPs:

2.1. **rs10968110**, chr 9, 27792965 bp

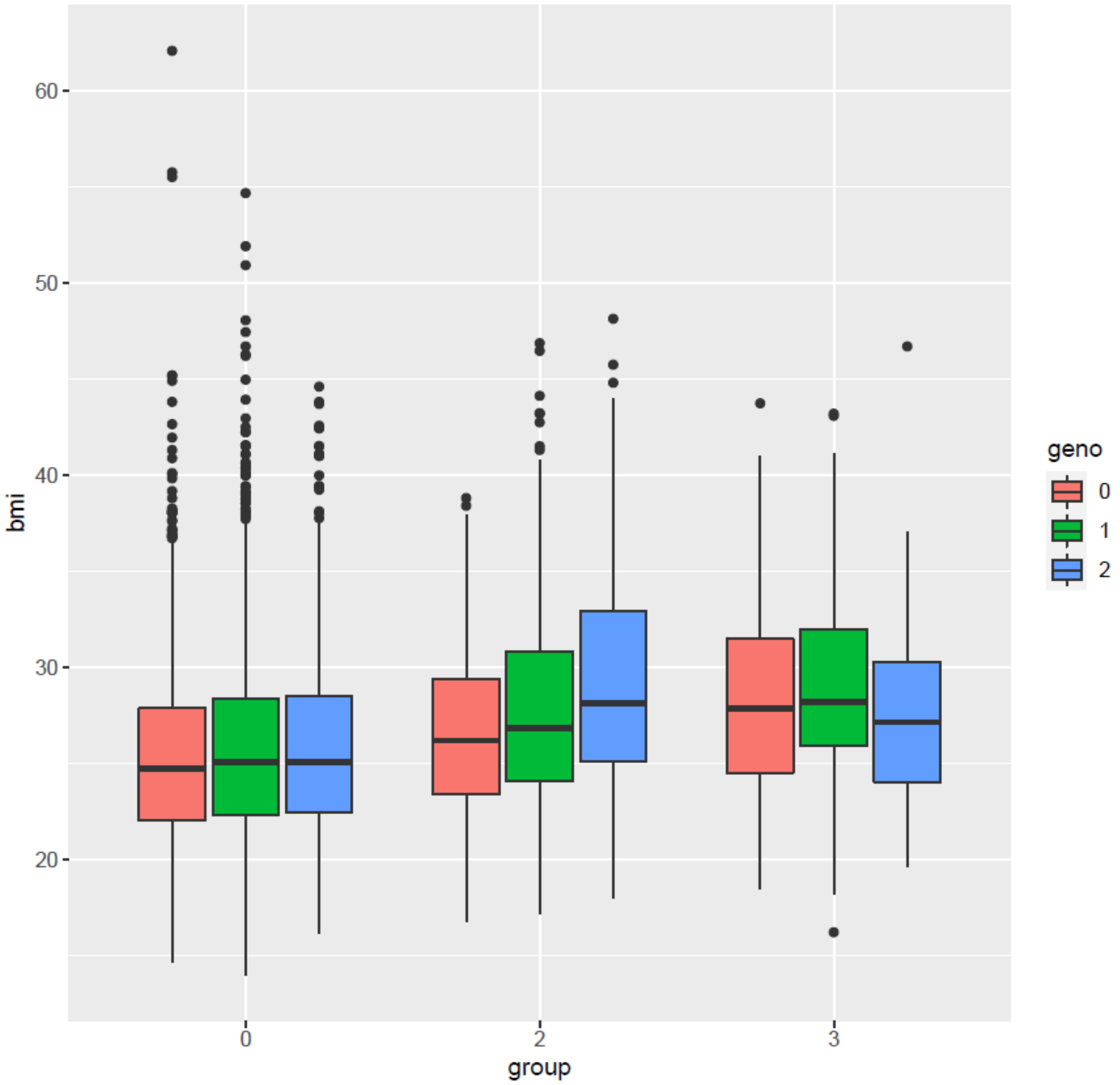

2.2. **rs4609724**, chr 13, 55666639 bp

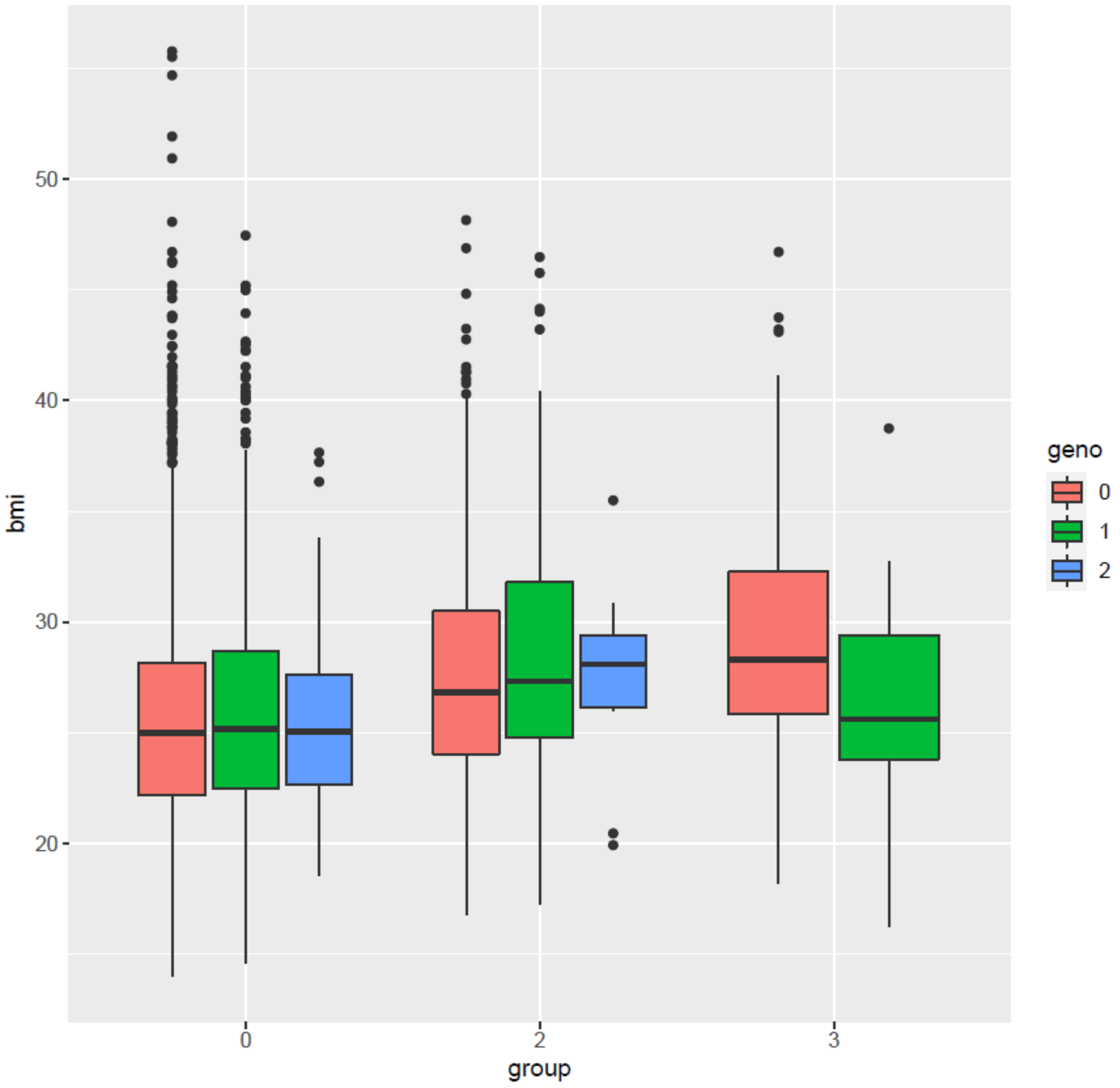

2.3. **rs4551082**, chr 5, 29620270 bp

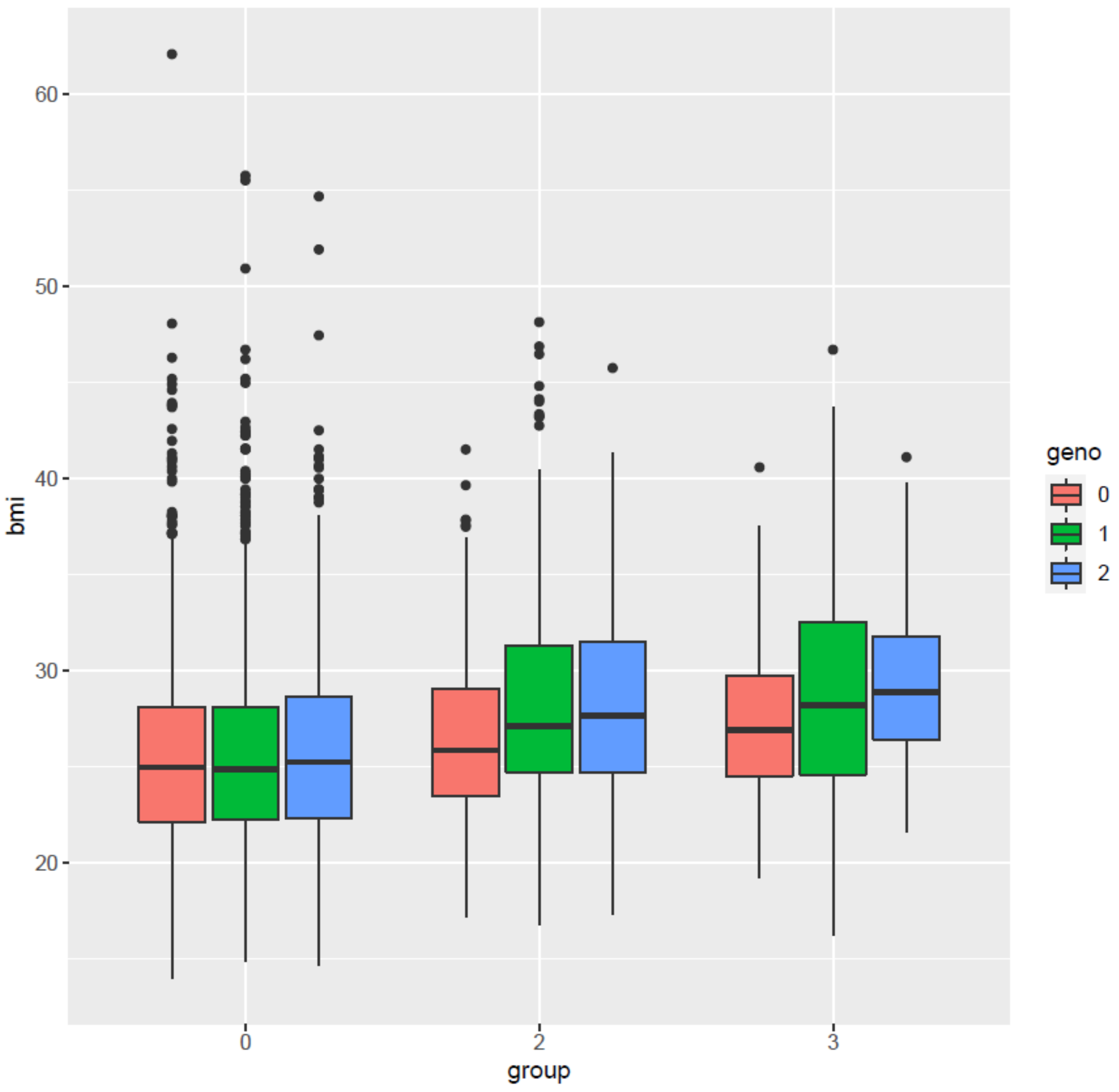

2.4. **rs9320269**, chr 6, 109032557 bp

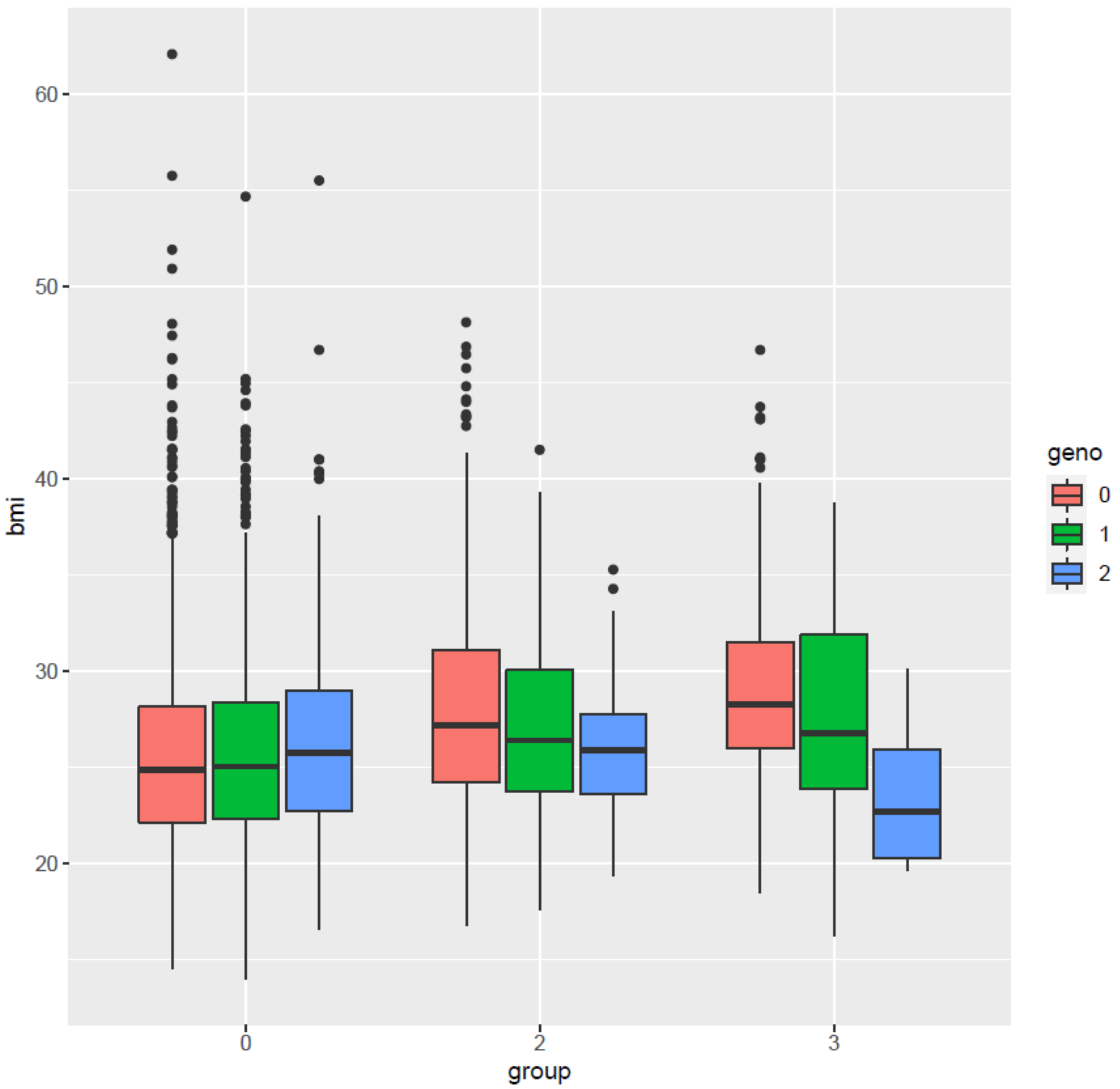

2.5. **rs2338833**, chr 5, 109032557 bp

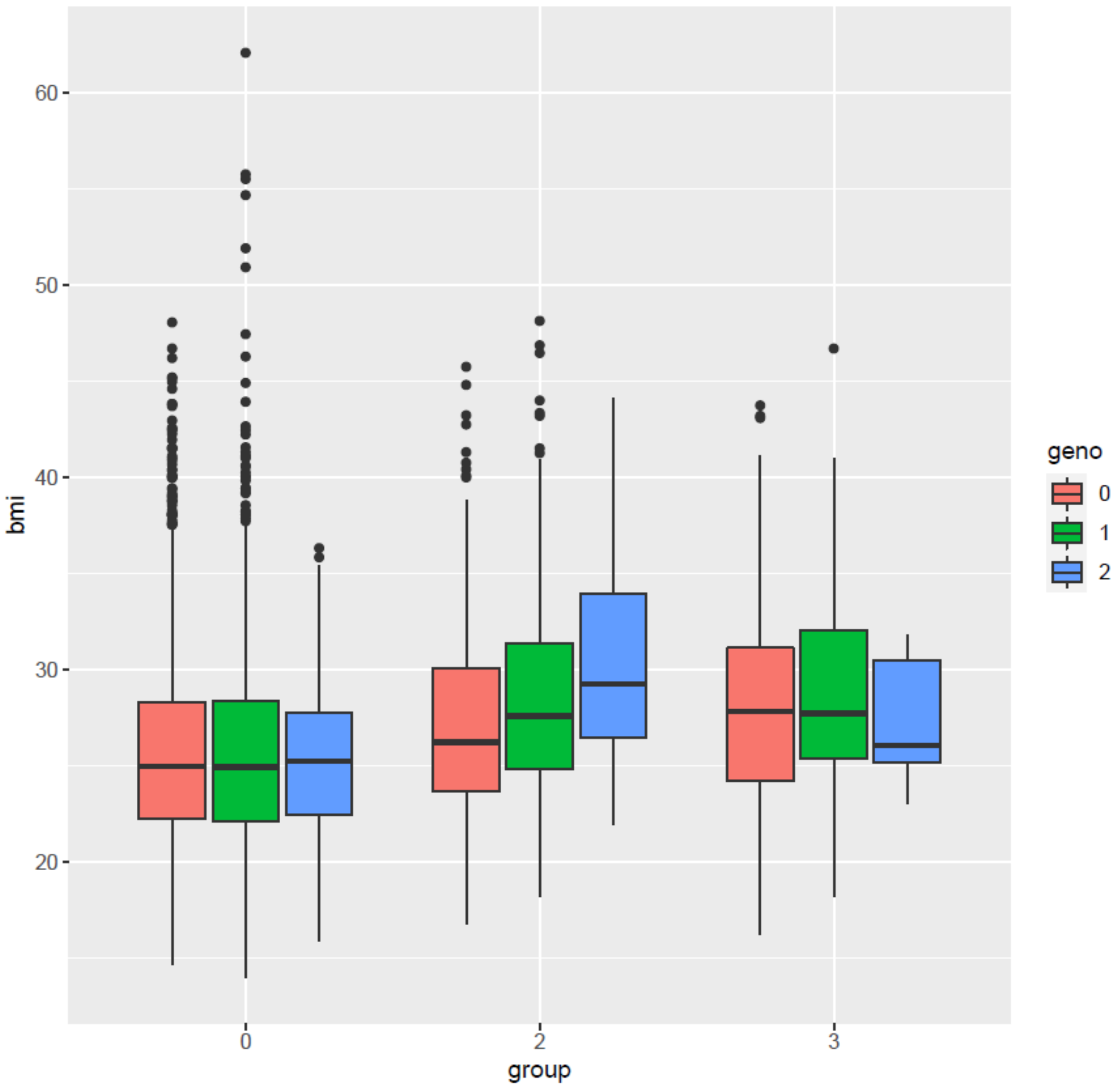

2.6. **rs1321529**, chr 6, 145601560 bp

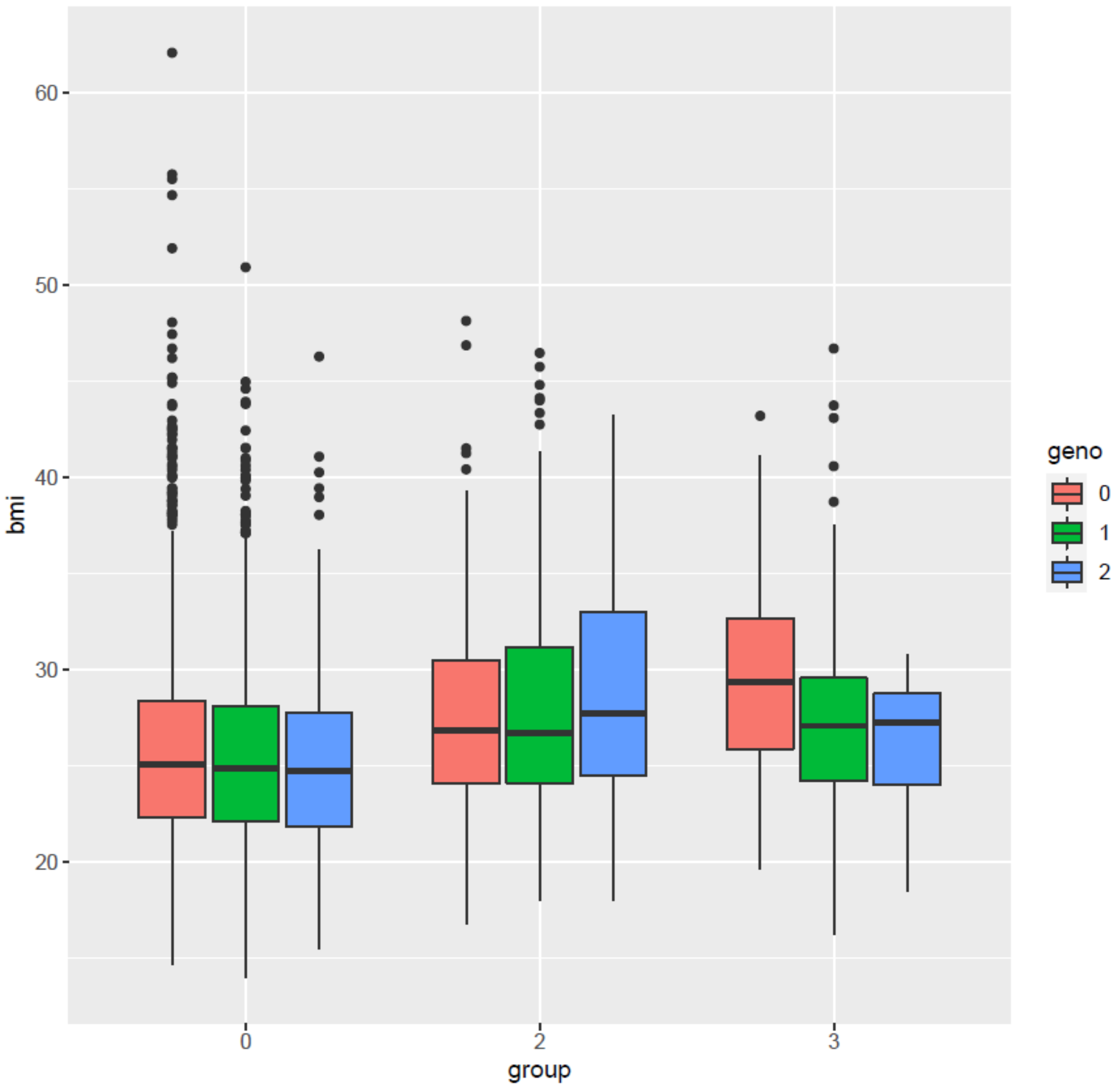

Supplement: Supplementary file 1 [file ijms-23-07396-s001.zip › Supplementary Figure S2.pdf]
